# Mesenchymal stem cell-derived exosome-educated macrophages alleviate systemic lupus erythematosus by promoting efferocytosis and recruitment of IL-17^+^ regulatory T cell

**DOI:** 10.1186/s13287-022-03174-7

**Published:** 2022-09-24

**Authors:** Mingchao Zhang, Takerra K. Johnson-Stephenson, Weiran Wang, Yang Wang, Jing Li, Limin Li, Ke Zen, Xi Chen, Dihan Zhu

**Affiliations:** 1 State Key Laboratory of Pharmaceutical Biotechnology, Jiangsu Engineering Research Center for MicroRNA Biology and Biotechnology, Nanjing University School of Life Sciences, Nanjing, Jiangsu China; 2 State Key Laboratory of Natural Medicines, School of Life Science and Technology, China Pharmaceutical University, Nanjing, Jiangsu China; 3 National Clinical Research Center of Kidney Diseases, Jinling Hospital, Nanjing University School of Medicine, Nanjing, Jiangsu China; 4 PRIME Education, Fort Lauderdale, FL USA; 5 Cardiovascular Research Institute, Morehouse School of Medicine, Atlanta, GA USA; 6 Department of Physiology, Shihezi University Medical College, Shihezi, Xinjiang China

**Keywords:** Systemic lupus erythematosus, Exosome, Macrophage, microRNA, Regulatory T cell

## Abstract

**Background:**

Anti-inflammatory polarized macrophages are reported to alleviate systemic lupus erythematosus (SLE). Our previous studies have demonstrated that exosomes from adipose-derived stem cells promote the anti-inflammatory polarization of macrophages. However, the possible therapeutic effect of exosomes from stem cells on SLE remains unexplored.

**Methods:**

Exosomes were isolated from the conditioned medium of bone marrow-derived mesenchymal stem cells using ultrafiltration and size-exclusion chromatography and were identified by nanoparticle tracking analysis and immunoblotting of exosomal-specific markers. Macrophages were collected from the MRL/lpr mouse kidney. The phenotype of macrophages was identified by immunoblotting for intracellular markers-inducible nitric oxide synthase (iNOS) and arginase-1 (Arg-1), and flow cytometry for macrophage markers F4/80, CD86, CD206, B7H4, and CD138. Pristane-induced murine lupus nephritis models were employed for in vivo study.

**Results:**

When macrophages from the kidney of the MRL/*lpr* mice were treated with exosomes from bone marrow-derived mesenchymal stem cells (BM-MSCs), the upregulation of CD206, B7H4, CD138, Arg-1, CCL20, and anti-inflammatory cytokines was observed, which suggested that the macrophages were polarized to a specific anti-inflammatory phenotype. These anti-inflammatory macrophages produced low levels of reactive oxygen species (ROS) but had a high efferocytosis activity and promoted regulatory T (T_reg_) cell recruitment. Moreover, exosome injection stimulated the anti-inflammatory polarization of macrophages and increased the production of IL-17^+^ T_reg_ cells in a pristane-induced murine lupus nephritis model. We observed that exosomes from BMMSCs depleted of microRNA-16 (miR-16) and microRNA-21 (miR-21) failed to downregulate PDCD4 and PTEN in macrophages, respectively, and attenuated exosome-induced anti-inflammatory polarization.

**Conclusion:**

Our findings provide evidence that exosomes from BMMSCs promote the anti-inflammatory polarization of macrophages. These macrophages alleviate SLE nephritis in lupus mice by consuming apoptotic debris and inducing the recruitment of T_reg_ cells. We identify that exosomal delivery of miR-16 and miR-21 is a significant contributor to the polarization of macrophages.

**Graphical abstract:**

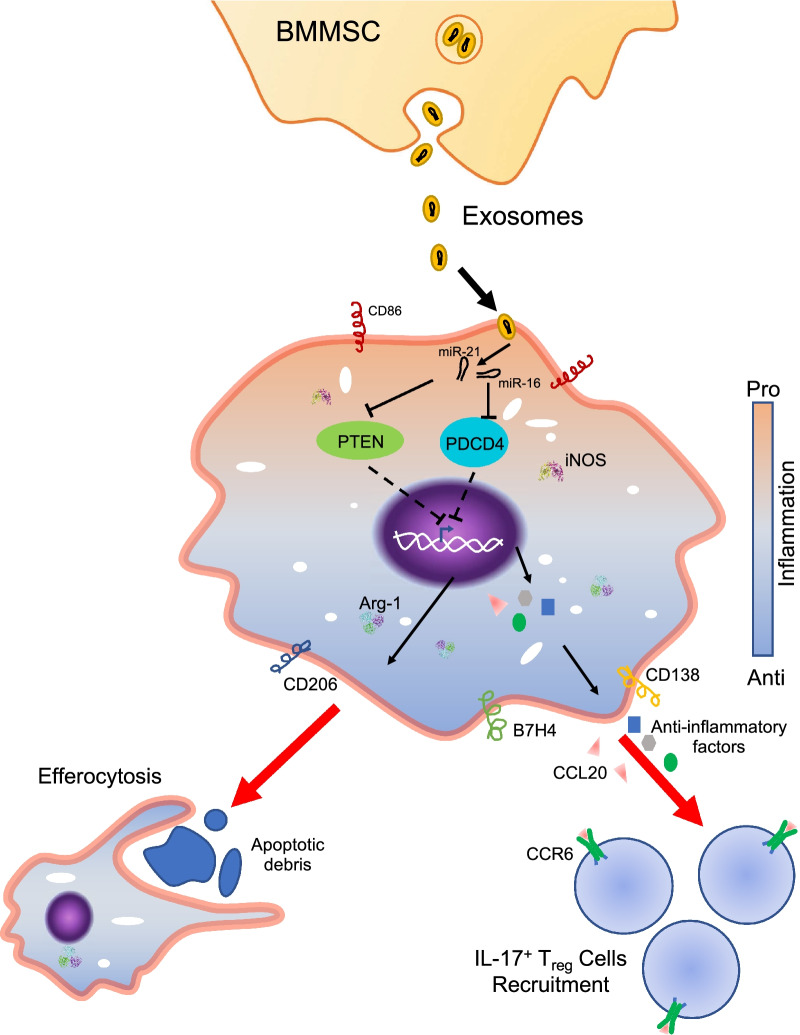

**Supplementary Information:**

The online version contains supplementary material available at 10.1186/s13287-022-03174-7.

## Introduction

Systemic lupus erythematosus (SLE) represents several lifelong heterogeneous diseases that can affect many organs, especially the skin, heart, and kidney [1]. The loss of tolerance, dysregulation of autoantibody production, and accumulation of apoptotic debris are primary causes of SLE [2, 3]. Uncleared apoptotic debris exposes nucleic acids to the immune system and evokes an inflammatory response by activating the Toll-like receptor (TLR) family and other nucleic acid recognition receptors [4]. The activated nucleic acid recognition receptors subsequently trigger pro-inflammatory cytokines production and provoke B cells and T cells recruitment and activation contributing to SLE progression. [5]

As one of the most important immune modulators, macrophages play a crucial role in immune response regulation [6]. Macrophages secrete many chemokines and cytokines which act as a critical bond that connects innate immunity with adaptive immunity [7]. Macrophages respond to environmental cues by polarizing into different phenotypes. Its remarkable plasticity gives them the ability to help eliminate foreign materials, aid in tissue repair, and regulate tissue homeostasis [8]. Under normal conditions, macrophages can recognize and dispose of apoptotic cells in a non-inflammatory way [9]. However, in patients diagnosed with SLE, macrophages' efferocytosis activity is reduced and leads to a buildup of apoptotic cells which then aggravated the pro-inflammatory polarization of macrophages [10]. Hallmarks of a pro-inflammatory response are the release of cytokines and chemokines that contribute to B cells and T cells' recruitment and activation, thereby accelerating SLE development [11]. The role of anti-inflammatory polarized macrophages in autoimmune diseases has been emphasized [12]. When macrophage's phenotype shifts from pro-inflammatory to anti-inflammatory, it increases the secretion of anti-inflammatory cytokines, accelerated apoptotic clearance, and increases T_reg_ cell recruitment which overall contributes to the relief of autoimmune diseases, like SLE [13–15].

Exosomes are a type of extracellular vesicle that transports bioactive cargo (protein, DNA, and RNA) from donor cells to distant cells and modulates target cell functions [16]. Exosomes from stem cells are reported to promote macrophage polarization. We discovered that exosomes from adipose-derived stem cells encourage macrophage anti-inflammatory polarization [17]. Also, exosomes from BMMSCs decrease the pro/anti-inflammatory ratio in models of lung cancer [18]. MiRNAs are small noncoding RNAs that participate in many biological processes, including macrophage polarization [19]. It has been verified that miRNAs delivered by exosomes from stem cells can induce anti-inflammatory polarization. [17]

This study demonstrates that exosomes derived from BMMSCs promote an anti-inflammatory polarization of macrophages collected from SLE mice. Exosomes derived from BMMSCs prime macrophages for high efferocytosis activity, anti-inflammatory cytokines secretion, and T_reg_ cell recruitment/differentiation. Exosomal miR-16 and miR-21 contribute to the effect of exosomes by targeting PDCD4 and PTEN, respectively.

## Material and methods

### Animals

Female C57BL/6 mice and MRL/*lpr* mice at age 6–8 weeks were purchased from The Jackson Labs (Bar Harbor, USA). Mice were placed in the SPF facility of the animal center of Nanjing University School of Life Sciences and reared at room temperature 25 ± 2 ℃, relative humidity 65 ± 2%, and a 12 h light / dark cycle. The mice were reared in an animal facility for one week before the experiment.

Mouse nephritis model was induced in an 8-week-old female C57BL/6 mouse by a single intraperitoneal injection of 500 μl of pristane oil (2,6,10,14-tetramethylpentadecane, Sigma-Aldrich, USA).

### Macrophage isolation from MRL-*lpr* mice kidney

Kidneys were processed as previously described [20], and the cell pellet was resuspended in D-hanks containing 0.5% BSA and 2 mM EDTA. Anti-F4/80 coated MACS beads (Miltenyi, Germany) were used to sort macrophages.

### Flow cytometry analysis for macrophages

Macrophages were resuspended and blocked for 30 min in D-hanks with 2% BSA. The cells were then incubated with 1:500 diluted FITC-anti-F4/80, or FITC-anti-CD206 and PE-anti-CD86, or FITC-anti-B7H4 or FITC-anti-CD138 antibody (BioLegend; San Diego, CA) for 45 min (room temperature and protected from light). The cells were then washed with D-hanks twice and resuspended in D-hanks with 2% BSA for flow cytometry analysis. The cells incubated with FITC-Isotype or PE-Isotype (BioLegend) were used as the isotype control. The gating strategy is shown in Additional file 1: sFig 1A.

### Western blot analysis

Cells were washed with PBS twice and lysed. Protein samples were extracted and then separated on a 4–12% precast Bis–Tris gel (Thermo Fisher Scientific). Later proteins were transferred to polyvinylidene difluoride (PVDF, Rancho Palos Verdes, USA) membranes. The membrane was blocked for 1 h in TBST (with 5% nonfat milk) and incubated with primary antibodies overnight at 4 ℃. Proteins were evaluated by using primary anti-iNOS and anti-Arg-1 (Cell Signaling, MA) antibodies. Membranes were washed with TBST 3 times (a total of 45 min). The washed membranes were incubated with second antibodies for 1 h, at room temperature. Enhanced chemiluminescence reagents were used to illuminate protein and ImageQuant ™ LAS 4000 Luminescent Image Analyzer (GE Healthcare; Chicago, IL) was used to expose protein bands.

### ELISA assays

The conditioned medium (CdM) was collected to evaluate the concentration of cytokines. Levels of certain cytokines, including IFN-*γ*, IL-1*β* IL-6, IL-12, GM-CSF, IL-10, TGF-*β*, and M-CSF, were evaluated by using ELISA kits (R&D Systems). The procedures for the cytokine and growth factor assays were carried out according to the manufacturer's instructions. The Molecular Devices SpectraMax spectrophotometer (Marshall Scientific, Hampton, NH) was employed to measure the concentration of cytokines.

### Detection of reactive oxygen species

ROS was detected by using Amplex Red Hydrogen Peroxide/Peroxidase Assay Kit (Molecular Probes, Grand Island, NY) according to the manufacturer’s instructions. Briefly, the macrophage-conditioned medium was collected, and the absorbance was checked by using the Molecular Devices SpectraMax spectrophotometer. The results were normalized to a standard curve.

### Phagocytosis assays

Mouse kidney epithelial cells (TCMK-1) were incubated with heat shock (45 ℃, 10-min) and cultured for 4 h to induce apoptosis as described previously. The percentage of apoptotic cells was evaluated by flow cytometry. The apoptotic cells were washed with PBS twice and labeled with pHrodo Red (Life Technologies). Then, the 1 × 10 [6] cells were added to 1 × 10^6^ macrophages to determine the phagocytotic capacity. After, being incubated for 2.5 h at 37 ℃, the macrophages were washed three times with ice-cold D-hanks and stained with FITC rat anti-mouse CD11b antibody (BioLegend, USA) for 45 min at 4 ℃. Then, the cells were harvested and examined by flow cytometry.

### Flow cytometry analysis for T cells

The cell suspension was blocked for 30 min in D-hanks with 2% BSA. The cells were then incubated with 1:500 diluted FITC-anti-Foxp3, or PE-anti-CD3, or PE/APC-anti-CD4, or APC-anti-IL-17 antibody (BioLegend; San Diego, CA) for 45 min (room temperature and protected from light). The cells were then washed with D-hanks twice and resuspended in D-hanks with 2% BSA for flow cytometry analysis. The cells incubated with FITC-Isotype or PE-Isotype or APC-Isotype (BioLegend) were used as the isotype control. The gating strategy is shown in Additional file 1: sFig 1B.

### Cell culture

The Mouse Bone Marrow-derived Mesenchymal Stem Cells were purchased from Cyagen (CA, USA). The cells were cultivated in an OriCell Mouse MSC Growth Medium (Cyagen) containing 100 units/ml of penicillin and 100 μg/ml of streptomycin at 37 ℃, 5% CO_2_. The medium was changed to fresh medium every 2–3 days.

### Exosome isolation

Exosomes were isolated from the BM-MSC medium by qEV exosomes isolation kit (iZON Science; MA, USA) according to the manufacturer’s protocol. Briefly, the conditioned medium was collected and centrifuged at 2,500 × g for 20 min to remove unattached cells and debris. A 100 kDa molecular weight cutoff in a centrifugal filter (Millipore Sigma; MA, USA) was employed to concentrate the medium. The concentrated medium was transferred to a size-exclusion chromatography qEV unit. The liquid that contained exosomes was collected and then reconcentrated by using a 10 kDa molecular weight cutoff centrifugal filter (Millipore Sigma).

The isolated exosomes were lysed, and the concentration of total proteins was measured by using a BCA Protein Assay Kit (Thermo Fisher Scientific, NJ, USA) according to the manufacturer’s instructions.

### Nanoparticle tracking analysis (NTA)

An LM10 NTA device (Malvern; Amesbury, UK) was used according to the manufacturer’s instructions to analyze the characteristics of microparticles that were isolated.

### Exosome uptake

Exosomes were labeled by using an ExoGlow-Protein EV Labeling Kit (Green, System Biosciences; Palo Alto, CA) according to the manufacturer’s instructions. Briefly, the exosomes were incubated with the green fluorescent dye at 37 ℃ for 20 min and then incubated at 4 ℃ overnight. The next day, the labeled exosomes were isolated and resuspended in the macrophage growth medium. Macrophages were incubated in the medium that contained 50 µg/ml exosomes for various times (3 h, 6 h, 12 h, or 24 h). The macrophages were harvested and incubated with a 1:10,000 diluted Hoechst 33,342 (Thermo Fisher Scientific) to label the nucleus. The fluorescent pictures were taken under fluorescent microscopy (Olympus DP70, Japan). For flow cytometry analysis, the cells were collected and analyzed by using a Guava easyCyte flow cytometer (Millipore Sigma). The results were further analyzed by using FlowJo software.

### Pathological scoring

The glomerular mesangial expansion was scored as follows: 0, no any expansion; 1, 1–10% glomeruli with mild expansion; 2, 11–25% glomeruli with moderate expansion; 3, 26–50% glomeruli with severe expansion; 4, 50% glomeruli being sclerotic. Twenty glomeruli were examined and scored for each mouse. A pathologist who was blinded to the sample identities performed the evaluation and scoring.

### Immunofluorescence staining

Sections of 5-mm thickness of mouse frozen kidney tissues were blocked with 10% FBS and incubated with primary antibodies labeled with FITC, rabbit polyclonal anti-IgG, IgA, IgM, C3, and C1q (DAKO, USA). The images were captured under the Leica microscope (DM5000B).

For IgG, IgA, IgM, C3, and C1q a pathologist blinded to the sample identities examined the glomeruli and scored staining intensity by 0 (no staining), 1 (weak staining), 2 (positive staining), and 3 (strongly positive).

### CCL20 analysis

The conditioned medium (CdM) of macrophage was collected to determine the concentration of CCL20 using ELISA kits (R&D Systems). The procedures were carried out according to the manufacturer's instructions. The Molecular Devices SpectraMax spectrophotometer (Marshall Scientific, Hampton, NH) was employed to measure the concentration of CCL20.

### Naïve T cell isolation

Mouse naïve T cell was isolated from the spleen by using Mouse Naïve CD4^+^ T Cell Isolation Kit (R&D Systems) according to the manufacturer's instructions. Briefly, the mouse spleen was teased apart to generate a single cell suspension in D-Hanks’ supplemented with 10% bovine serum. The cells were washed and resuspended in cold 1X MagCellect Buffer at 1 × 10^8^ concentration. Then, the cells were incubated with Mouse Naïve CD4^+^ T Cell Biotinylated Antibody Cocktail,

### RNA isolation and qRT-PCR of miRNAs

Total RNA was extracted from cells or exosomes using TRIzol Reagent (ThermoFisher) according to the manufacturer’s instructions. Quantitative RT-PCR was performed using TaqMan miRNA probes (Applied Biosystems, USA) according to the manufacturer’s instructions. Briefly, total RNA was reverse transcribed to cDNA using AMV reverse transcriptase (ThermoFisher) and a stem-loop RT primer (Applied Biosystems) or RT primer. Real-time PCR was performed using a TaqMan PCR kit and an Applied Biosystems 7900 Sequence Detection System (Applied Biosystems). All reactions, including no-template controls, were run in triplicate. After the reaction, the CT values were determined using fixed threshold settings. miRNA expression in cells or tissues is normalized to U6 snRNA or Cel-miR-39.

### Transduction of recombinant lentivirus

The procedures practiced here followed the National Institutes of Health guidelines for recombinant DNA research. All recombinant lentiviruses used in this study were purchased from the AMSBIO (Abingdon, UK). For transduction, macrophages were incubated with the Lenti/PREmiR-16 (PREmiR-16) and Lenti/PREmiR-21 (PREmiR-21) or Lenti/PREmiR-Cont (PREmiR-Cont) at a multiplicity of infection (MOI) of 5. BMMSCs were incubated with Lent/ZIPmiR-16 (ZIPmiR-16) and Lent/ZIPmiR-21 (ZIPmiR-21) to deplete miR-16 and miR-21 levels in the cells. Lent/ZIPmiR-Cont (ZIPmiR-Cont) was used as control.

### Data analysis

All values are shown as the means ± SDs. All experiments were repeated at least 4 times (*n* = 4) unless otherwise noted. Student’s *t* test analysis was performed to analyze all data. Only *p* < 0.05 was considered significant.

## Results

### Macrophages infiltrated in the kidney of MRL/***lpr*** mice are polarized to a B7H4^−^ and CD138^lo^ pro-inflammatory phenotype

**Fig. 1:**
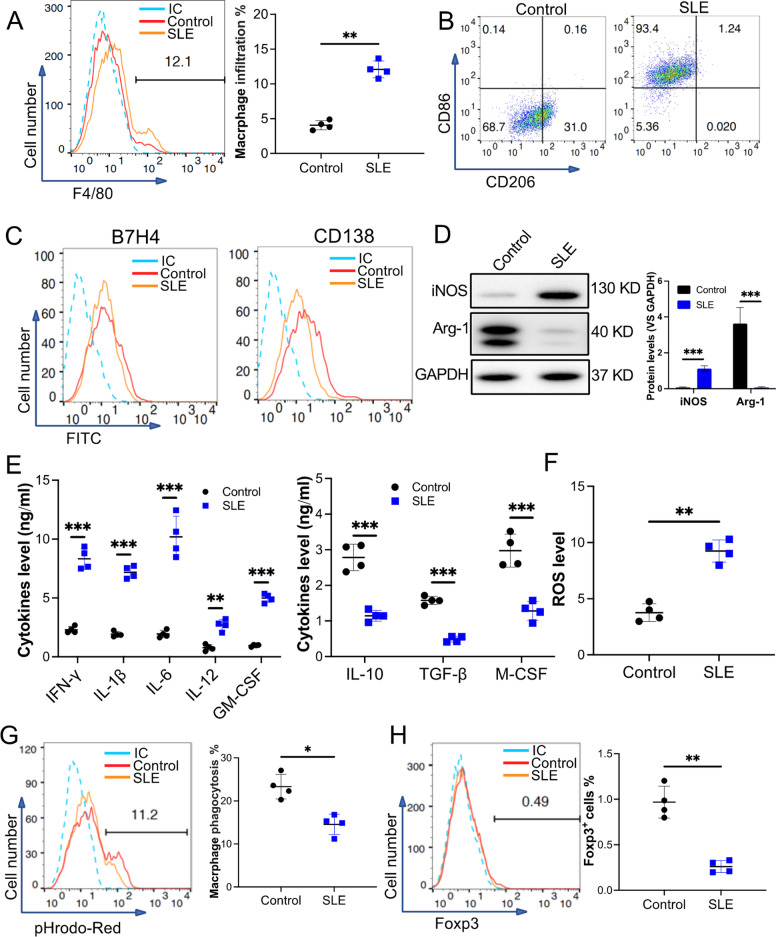
Characterization of macrophages from the kidney of 18-week-old MRL/*lpr* mice. The mice (10 mice) were killed and kidneys were collected. F4/80^+^ pan macrophages were sorted. Eighteen-week-old C57 BL/6 mice (10 mice) were used as the control. **A** The F4/80^+^ macrophage infiltration in the kidneys. **B** and **C**Macrophage surface markers, CD86 and CD206 (B) or B7H4 and CD138 (C), were checked by using flow cytometry. **D** Macrophage intracellular markers, iNOS and Arg-1, were examined by using immunoblotting. GAPDH was used as a control (*n* = 3). **E** The pro- and anti-inflammatory cytokines secreted from the macrophages were measured using ELISA. **F** The ROS level of the macrophages. **G** The efferocytosis of pHrodo Red-labeled apoptotic TCMK-1 cells (pHrodo Red) by the macrophages. **H** The Foxp3^+^ Treg infiltration in the kidneys was analyzed by using flow cytometry. Data are expressed as mean ± SD of *n* = 4, unless specified. *p < 0.05, **p < 0.01 and ***p < 0.001. IC, isotype control. Control, the cells collected from C57 BL/6 mice. SLE, the cells collected from MRL/*lpr* mice

There is a known role of macrophages in the development of SLE [21]. To further study the phenotype of macrophages in SLE mice, macrophages from the kidney of 18-week-old MRL/*lpr* mice were collected. Our results demonstrated that infiltration of F4/80^+^ macrophages in SLE mice was higher than in the wild-type mice (control) (Fig. 1A). Flow cytometry analysis revealed that macrophages collected from SLE mice presented higher levels of CD86, a pro-inflammatory polarization marker, but presented low levels of CD206, an anti-inflammatory polarization marker, when compared to macrophages from control mice (Fig. 1B). Both groups of macrophages failed to express B7H4 and CD138 (Fig. 1C). Immunoblot results indicated increased protein levels of iNOS while the level of Arg-1 decreased in the macrophages from SLE mice (Fig. 1D). These results suggest that macrophages in the kidney of MRL/*lpr* mice are polarized to a specific phenotype defined as CD86^hi^CD206^−^B7H4^−^CD138^lo^iNOS^hi^Arg-1^−^. Cytokine analysis of the conditioned medium of macrophages collected from SLE mice exhibited upregulation of pro-inflammatory cytokines IFN-*γ*, IL-1*β*, IL-6, IL-12, and GM-CSF. In contrast, anti-inflammatory cytokines IL-10, TGF-*β*, and M-CSF were downregulated in these macrophages (Fig. 1E). Also, reactive oxygen species (ROS) are active contributors to pro-inflammatory conditions; we found that ROS levels were upregulated in the macrophages from SLE mice (Fig. 1F). Many studies support that an accumulation of apoptotic cells plays a crucial role in SLE progression. Efferocytosis activity analysis demonstrated that macrophages from SLE mice exhibit a lower efferocytosis activity than control mice (Fig. 1G). T_reg_ cells are potent immune suppressors and were observed to be downregulated in SLE patients. Our results demonstrated that Foxp3^+^ T cells (T_reg_) decreased in the SLE mouse kidney (Fig. 1H). However, the upregulation of T helper (Th) 17 cells that facilitate inflammation and SLE development was detected in the SLE mouse's kidney (Additional file 1: sFig 2A). Further, the accumulation of pro-inflammatory macrophages and Th17 cells resulted in the downregulation of anti-inflammatory cytokines but the upregulation of pro-inflammatory cytokines in the serum from SLE mice (Additional file 1: sFig 2B and 2C). These data suggest that macrophages from MRL/*lpr* mice are polarized to a pro-inflammatory phenotype and cause a reduced efferocytosis activity and failed apoptotic cell clearance that further induces the steady deterioration of SLE.

### Exosomes from BMMSCs alleviated mouse SLE nephritis

**Fig. 2:**
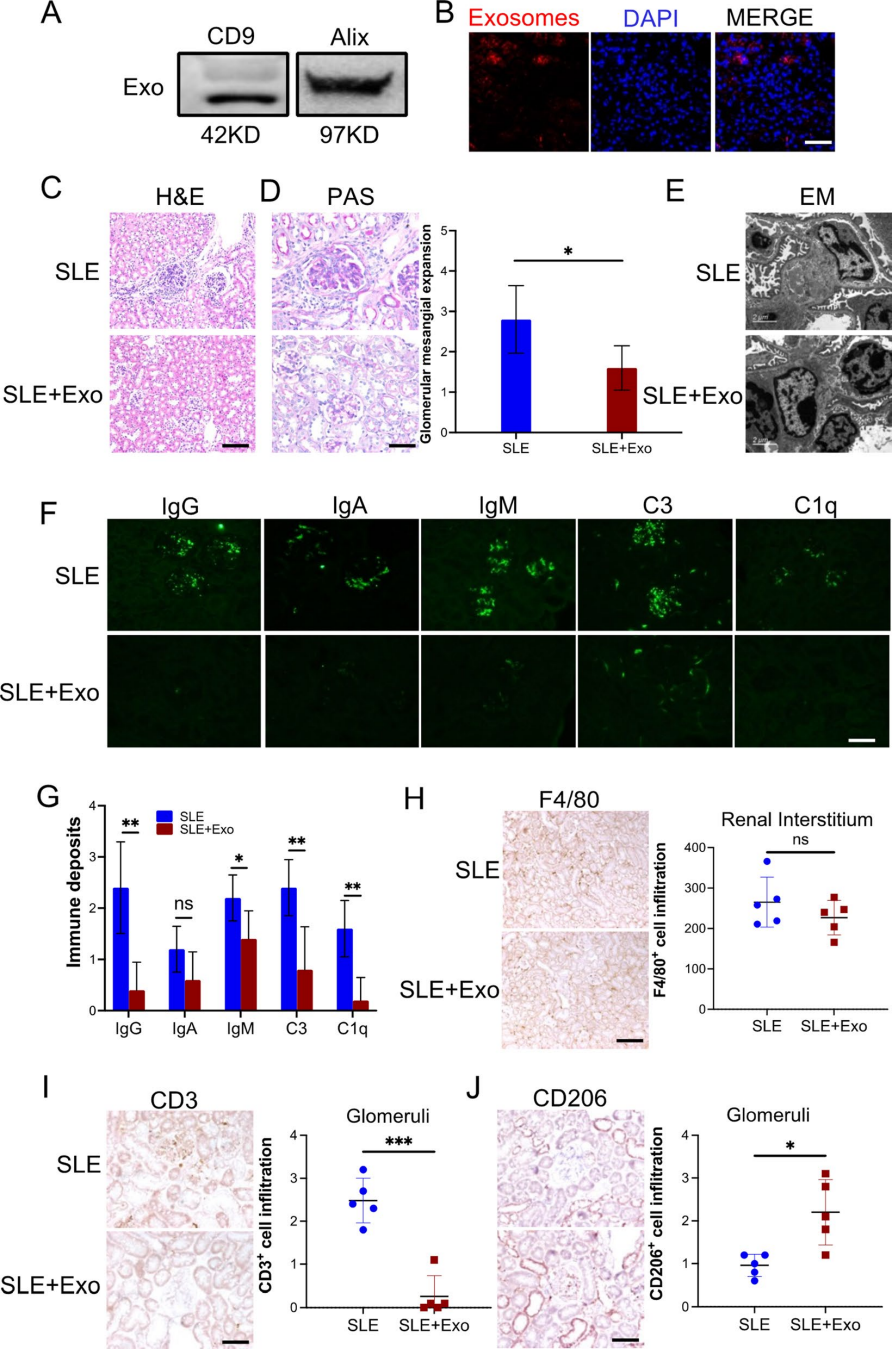
Exosome from BMMSCs attenuated SLE nephritis in vivo. MRL/lpr mice (18-week-old) were tail vein injected 100 µg exosomes on day 0, 3, 7 (SLE + Exo, *n* = 5); PBS was used as control (SLE, *n* = 5). On day 10, all mice were killed and the kidneys were harvested to evaluate the therapeutic effect of exosome in vivo. **A** The exosomal markers TSG101 and Alix were determined via immunoblotting (*n* = 3). **B** The accumulation of the DILC-18 labeled exosomes (red) in the kidney (scale bar: 100 µm). **C** Immune cell infiltration was examined by H&E stain (scale bar: 100 µm). **D** Glomerular mesangial expansion was checked through PAS stain (scale bar: 50 µm). **E** The level of dense deposits in glomerular mesangial was determined by electron microscope (EM). **F** and ** G** Immune deposits level in glomerular mesangial and endocapillary (green, scale bar: 100 µm). **H** Macrophage (F4/80^+^) infiltration in renal interstitium of kidney from exosome-treated mice (scale bar: 100 µm). **I** CD3^+^ T cell infiltration in glomeruli (scale bar: 50 µm). **J** CD206^+^ macrophage infiltration in glomeruli (scale bar: 50 µm). Data are expressed as mean ± SD of *n* = 5, unless specified. *p < 0.05, **p < 0.01 and ***p < 0.001. ns, nonsignificant

Exosomes are microparticles secreted from cells. In this study, we utilized a nanoparticle tracking analysis to determine the characteristics of the exosomes that were isolated from BMMSCs. The isolated particles (~ 40 nm) fell within the normal size distribution of exosome (Additional file 1: sFig 3A). Immunoblot analysis for known exosome markers, TSG-101 and Alix, was identified and detected (Fig. 2A). Green fluorescent-labeled exosomes were used to investigate uptake efficiency. Fluorescent images (Additional file 1: sFig 3B) and flow cytometry analysis (Additional file 1: sFig 3C) revealed that the internalization of exosomes into macrophages increased to 90% after 24 h. To study the role of exosomes from BMMSCs in alleviating SLE under pathophysiological conditions, 18-week-old MRL/*lpr* mice were tail vein injected exosomes from BMMSCs on day 0, day 3, and day 7; PBS was used as control. On day 10, the kidney, lung, liver, heart, and spleen were harvested from all mice. Our results showed that tail vein injected exosomes were significantly accumulated in the liver and kidney (Fig. 2B and Additional file 1: sFig 4). Results of H&E and PAS staining demonstrated that the exosome treatment downregulated immune cell infiltration in the renal interstitium (Fig. 2C) and reduced glomerular mesangial expansion (Fig. 2D); PBS was used as a control. Electron microscope observation and immunofluorescence further confirmed that exosome treatment lessened dense deposits (Fig. 2E) and immune deposits including IgG, IgM, C3, and C1q (Fig. 2F and 2G) in the glomerular mesangial and endocapillary, respectively. Interestingly, although there was no difference in the infiltration of macrophages in the kidney of mice that received exosomes or PBS (Fig. 2H and Additional file 1: sFig 5), T cell infiltration was significantly downregulated in the kidney of the exosome-treated mouse (Fig. 2I and Additional file 1: sFig 6). Moreover, exosome treatment promoted kidney-infiltrated macrophage M2-like polarization, which was defined by a high level of CD206 (Fig. 2J and Additional file 1: sFig 7). Altogether, our results demonstrated that exosomes from BMMSCs alleviated SLE nephritis in lupus mouse by increasing kidney accumulation of M2-like macrophage and reducing the kidney infiltration of T cells.

### Exosomes from BMMSCs polarized pro-inflammatory macrophage from the kidney of RL/*lpr* mice to a specific anti-inflammatory phenotype

**Fig. 3:**
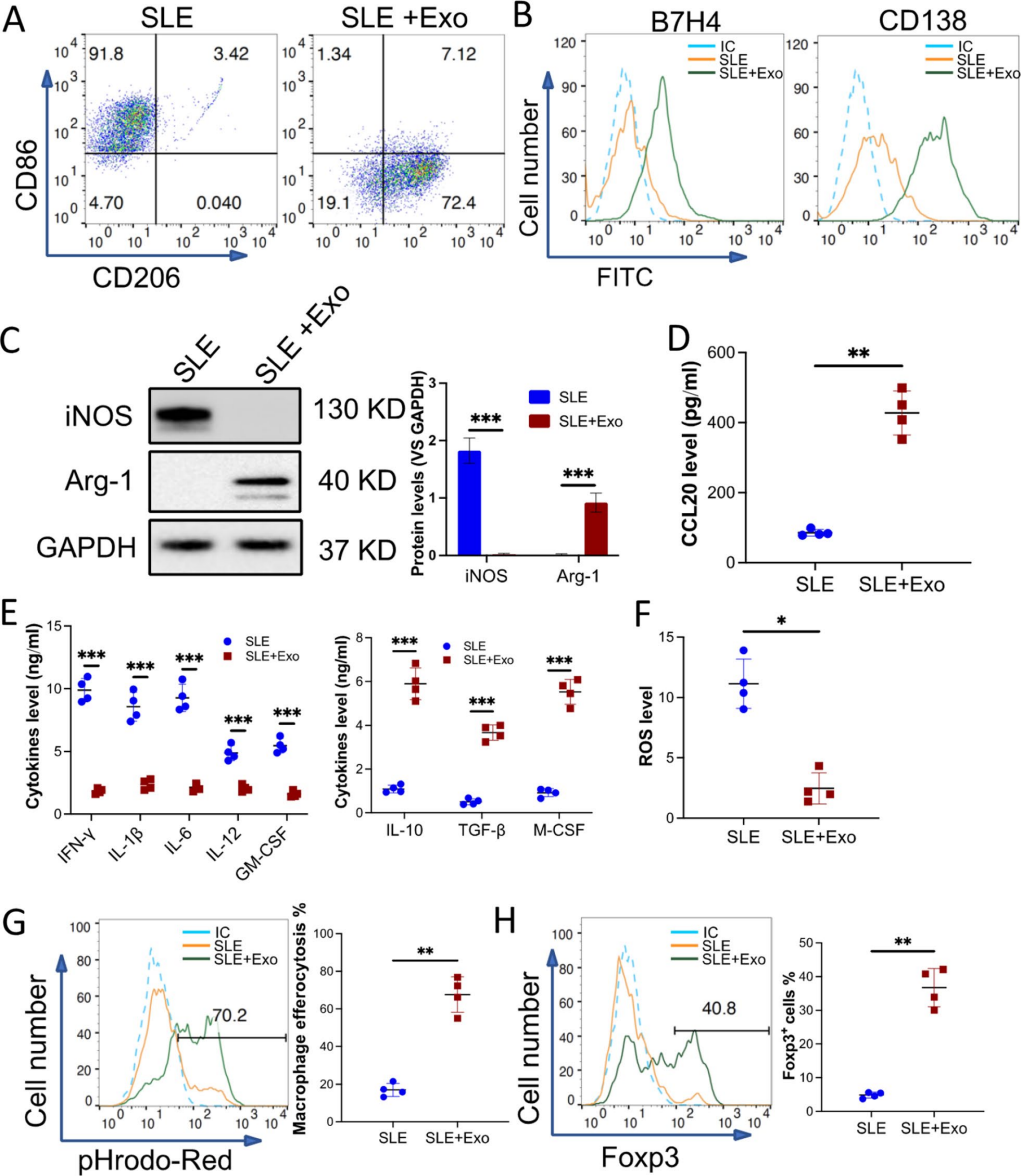
Macrophages from the kidney of MRL/*lpr* mice were polarized to a unique anti-inflammatory phenotype by exosomes from BMMSCs. Pan macrophages were sorted from the kidney of MRL/*lpr* mice (10 mice) then treated with 50 µg/ml exosomes from BMMSCs for 48 h in vitro (SLE + Exo), PBS treatment was used as control (SLE). **A** and **B**The phenotype of exosome-treated macrophage were evaluated. Surface markers CD86 and CD206 (A) or B7H4 and CD138 (B) were checked by using flow cytometry. **C** Macrophage intracellular markers, iNOS and Arg-1 were examined by using immunoblotting. GAPDH was used as a control. **D**and **E**The level of CCL20 (D) or pro- and anti-inflammatory cytokines (E) in the conditioned medium (CdM) of exosome-treated macrophage were detected by ELISA. **F** The ROS level of the macrophages. **G** The efferocytosis of pHrodo Red-labeled apoptotic TCMK-1 cells by the macrophages. **H** Naïve T cells were treated with CdM of macrophages. The percentage of Fox3p^+^ induced Treg **(**iTreg) was checked by flow cytometry. Data are expressed as mean ± SD of *n* = 4, unless specified. *p < 0.05, **p < 0.01 and ***p < 0.001. IC, isotype control

Exosomes, especially from stem cells, are reported to mediate many biofunctions including macrophage polarization [17]. To study the role of exosome from BMMSCs in macrophage polarization, macrophages were collected from 18-week-old MRL/*lpr* mice kidneys and incubated with 50 µg/ml exosomes derived from BMMSC for 3 days. Flow cytometry analysis demonstrated that CD86 on the macrophages was downregulated, while CD206 was upregulated (Fig. 3A). Moreover, compared to PBS-treated macrophages, exosome-treated macrophages expressed more B7H4 and CD138 (Fig. 3B). Additionally, exosome treatment downregulated iNOS but upregulated Arg-1 in the macrophages (Fig. 3C). Furthermore, the conditioned medium from macrophages treated with the exosomes was analyzed for their secretory content. ELISA results detected elevated levels of CCL20, a chemokine normally found ligated to CCR6 on T cells (Fig. 3D). Meanwhile, pro-inflammatory cytokines such as IFN-*γ*, IL-1*β*, IL-6, IL-12, GM-CSF were decreased, and anti-inflammatory cytokines including IL-10, TGF-*β*, and M-CSF were increased (Fig. 3E). We found that there was a significant reduction in reactive oxygen species (ROS) levels in exosome-treated macrophages, this further confirmed that macrophages had shifted to an anti-inflammatory phenotype (Fig. 3F). Again, an efferocytosis assay was used to determine the efferocytosis activity of macrophages. Exosome-treated macrophages efferocyted increasingly more apoptotic cells when compared to PBS-treated macrophages (Fig. 3G). More interestingly, conditioned medium collected from exosome-treated macrophages induced more Naïve T cells to differentiate into T_reg_ cells (Fig. 3 H). These results suggest that pro-inflammatory macrophages from SLE mice can be polarized to a defined anti-inflammatory phenotype, CD206^+^, Arg-1^+^, B7H4^+^, and CD138^+^ if pre-treated with exosomes. These macrophages secrete high levels of anti-inflammatory cytokines, efferocyte more apoptotic cells, and even evoke naïve T cells' transition to T_reg_ cells.

### Exosomal miR-16 and miR-21 contribute to exosome-induced anti-inflammatory polarization of macrophages

**Fig. 4:**
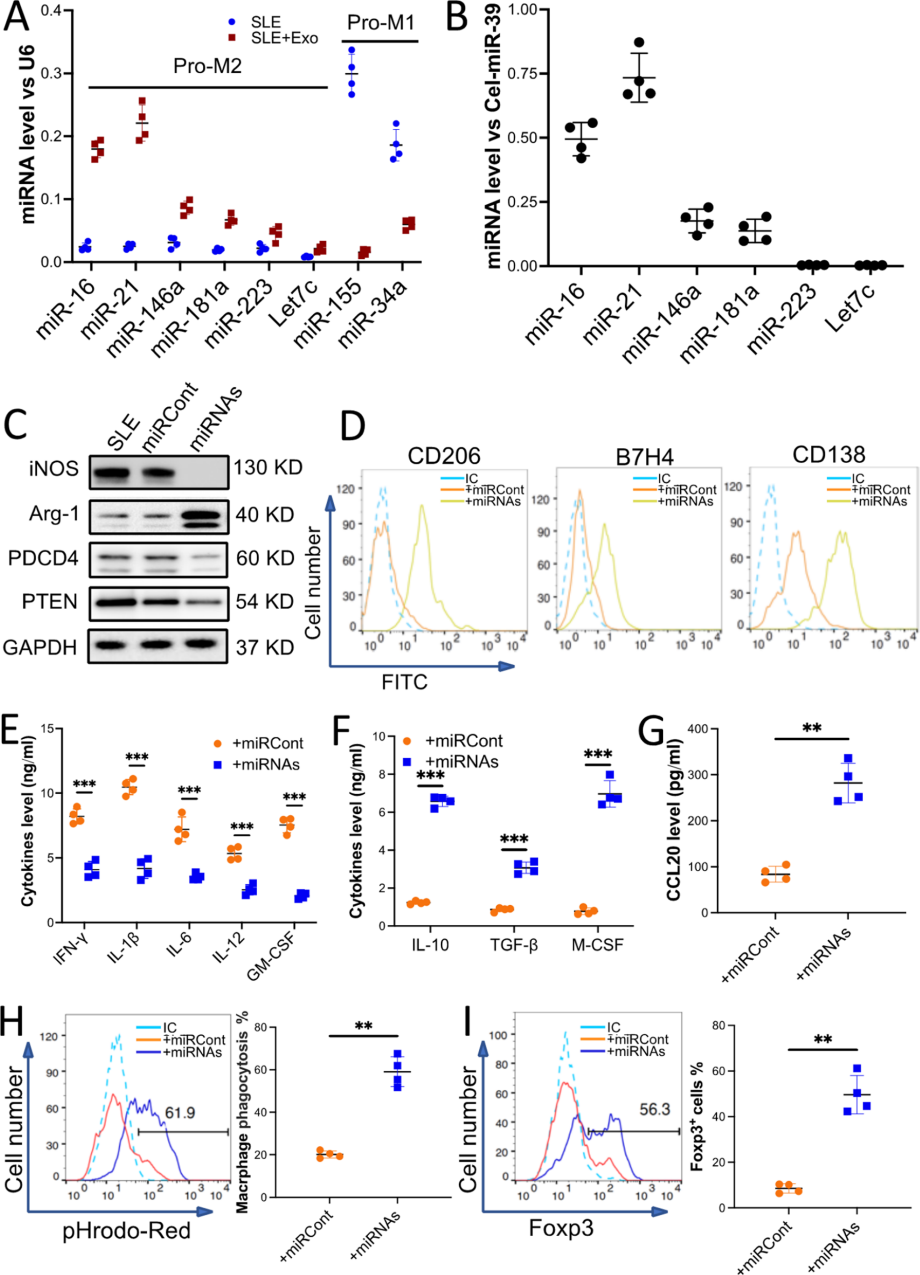
miR-16 and miR-21 enriched in the exosomes from BMMSCs contributed to the macrophage's unique anti-inflammatory polarization. Macrophages sorted from the kidneys of MRL/*lpr* mice (10 mice) were incubated with 50 µg/ml exosomes for 48 h (SLE + Exo), PBS treatment was used as a control (SLE). **A** The level of miRNAs that promote anti-inflammatory polarization (Pro-M2) or pro-inflammatory polarization (Pro-M1) in the macrophages with or without exosome treatment. **B** The relative level of miRNAs that promote macrophage anti-inflammatory polarization in the exosomes was calculated by qRT-PCR. Exogenous reference (Cel-miR-39) was added at exact amounts in each sample to estimate the efficiency of RNA extraction and reverse transcription (RT) reaction and to normalize the expression level of miRNAs in the exosome. **C–H** The macrophages were transduced with miRNA-Lentivirus to overexpress miR-16 and miR-21 (+ miRNAs). The miRNA-Lentivirus control was used as a control (+ miRCont). The levels of iNOS, Arg-1, PTEN, and PDCD4 in the miRNAs’ overexpressed macrophages were examined by immunoblotting analysis. GAPDH was used as a control (**C**). Macrophage surface markers CD206, B7H4, and CD138 (**D**) were checked by using flow cytometry. Macrophage-secreted pro- and anti-inflammatory cytokines (**E** and **F**) or chemokine CCL20 (**G**) was detected by ELISA. The efferocytosis of pHrodo Red-labeled apoptotic TCMK-1 cells by the macrophages (**H**). **I** Naïve T cells were incubated with CdM of macrophages that overexpressed miRNAs. The percentage of Fox3p^+^ iTreg was checked by flow cytometry. Data are expressed as mean ± SD of *n* = 4, unless specified. **p < 0.01 and ***p < 0.001. IC, isotype control

To understand the underlying molecular mechanisms of exosome-mediated macrophage polarization, total RNA from exosome-treated macrophages was isolated and subjected to miRNA profiling. Total RNA from PBS-treated macrophages was used as a control. The results of microarray analysis indicated that 20 miRNAs from exosome-treated macrophages were 2 times more upregulated when compared to PBS-treated macrophages (*p* < 0.05, Additional file 1: sFig 8). Among these upregulated miRNAs, 6 miRNAs were reported to promote macrophage anti-inflammatory polarization. The 6 miRNAs were validated using qRT-PCR analysis (Fig. 4A). In the context of this study, we naturally hypothesized that miRNAs aid in anti-inflammatory polarization of exosome-treated macrophage and these miRNAs may be delivered by exosomes. Interestingly, qRT-PCR results showed that miR-16 and miR-21 were enriched in the exosomes from BMMSCs (Fig. 4B).

It has been reported that miR-16 promotes macrophage anti-inflammatory polarization by targeting PDCD4 and miR-21 similarly does the same by targeting PTEN. To investigate the role of miR-16 and miR-21 in the SLE macrophage anti-inflammatory polarization, macrophages were transduced using a lentivirus-based miRNA overexpression vector to upregulate cellular miR-16 and miR-21. Macrophages transduced with lentivirus control were used as a control. After transduction, the levels of miR-16 and miR-21 were elevated significantly (Additional file 1: sFig 9A). Interestingly, immunoblot analysis demonstrated that the levels of iNOS, PDCD4, and PTEN decreased, but the level of Arg-1 increased in the macrophages that overexpressed miR-16 and miR-21 (Fig. 4C and Additional file 1: sFigure 9B). Further, miR-16 and miR-21 overexpression induced an increase in CD206, B7H4, and CD138 surface markers on the macrophages (Fig. 4 D). More important, there was a significant downregulation of pro-inflammatory cytokines (Fig. 4 E) and ROS (Additional file 1: sFig 9C), but upregulation of anti-inflammatory cytokines (Fig. 4 F) and chemokine CCL20 (Fig. 4 G) was observed in the miRNAs overexpressed macrophages. Results from the efferocytosis assay concluded that by overexpressing miR-16 and miR-21 in macrophages, it incurred increased efferocytosis of apoptotic cells than untransduced macrophages (Fig. 4H). Similarly, miRNA-overexpressed macrophages induced more production of T_reg_ cells (Fig. 4I).

**Fig. 5:**
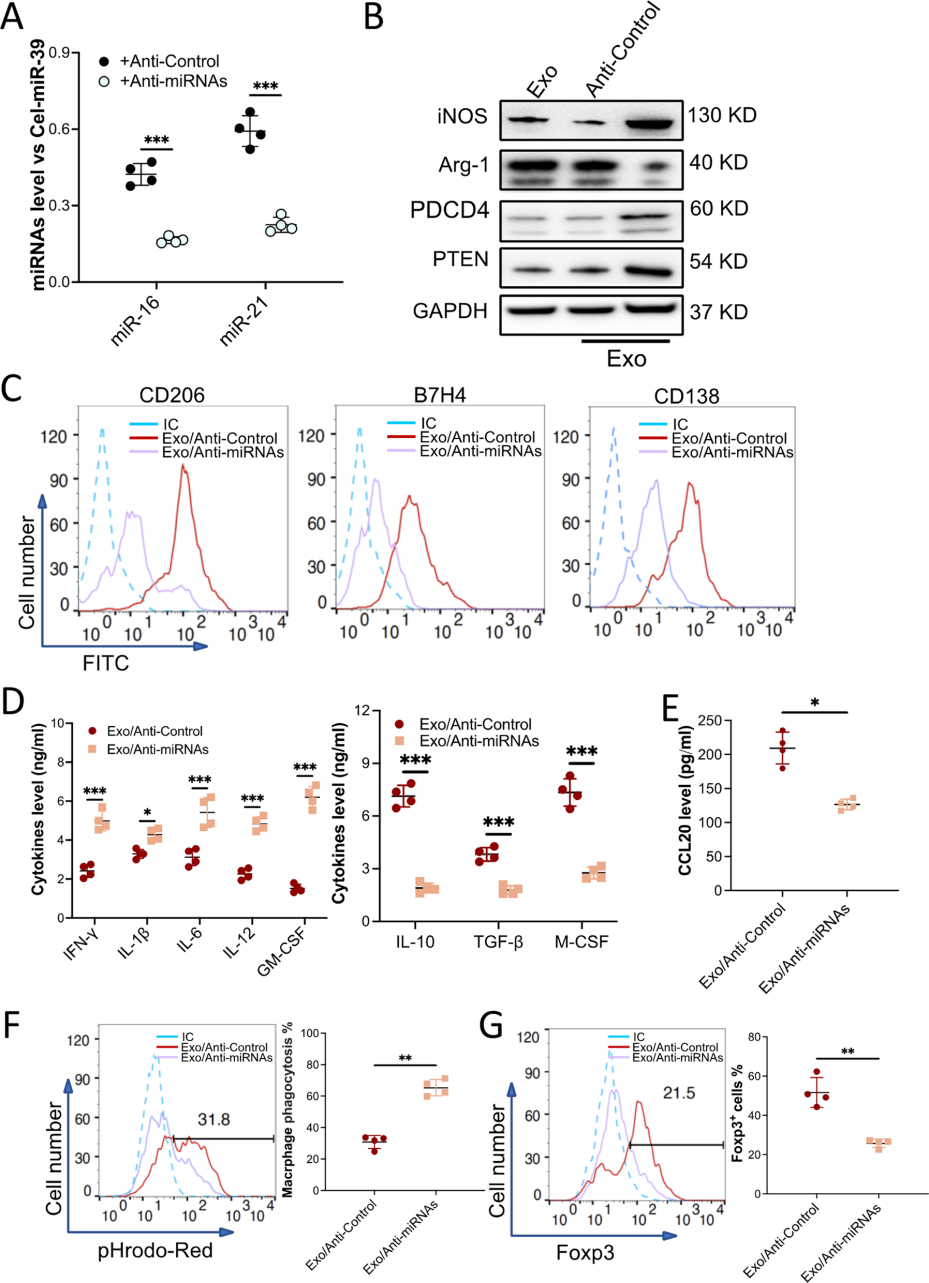
Exosome-induced macrophage polarization was attenuated when miR-16 and miR-21 were depleted in the exosomes. BMMSCs were transduced with Lentivirus/anti-miR-Control (+ Anti-Control) or with Lentivirus/anti-miR-16 and anti-miR-21 (+ Anti-miRNAs) to knock down miR-16 and miR-21. Exosomes from these cells were collected. **A** The level of miR-16 and miR-21 in the exosomes was validated with qRT-PCR. Cel-miR-39 was introduced as an exogenous reference for the normalization of miRNAs in the exosome. **B–F** Macrophages sorted from the kidney of MRL/*lpr* mice were treated with miR-16 and miR21-downregulated exosomes (Exo/Anti-miRNAs, *n* = 4). Exosome collected from BMMSCs that received Lentivirus/anti-miR-Control (Exo/Anti-miR-Control, *n* = 4) was used as a control. The levels of iNOS, Arg-1, PTEN, and PDCD4 in the macrophages were examined by immunoblotting analysis. GAPDH was used as controls (**B**, *n* = 3). Macrophage surface markers CD206, B7H4, and CD138 (**C**) were checked by using flow cytometry. Macrophage-secreted pro- and anti-inflammatory cytokines (**D**) or chemokine CCL20 (**E**) were detected by ELISA. The efferocytosis of pHrodo Red-labeled apoptotic TCMK-1 cells by the macrophages (**F**). **G** Naïve T cells were incubated with CdM of macrophages that were treated with the exosomes. The percentage of Fox3p^+^ iTreg was checked by flow cytometry. Data are expressed as mean ± SD of *n* = 4, unless specified. *p < 0.05, **p < 0.01 and ***p < 0.001. IC, isotype control

Next, we depleted miR-16 and miR-21 in exosomes. To do this, we isolated exosomes from BMMSCs that were transduced with a lentivirus-based anti-miRNAs vector; the level of miR-16 and miR-21 was downregulated in the exosomes (Fig. 5A). When macrophages were treated with the miRNAs-knockdown exosomes, the exosomes treatment induced upregulation of miR-16 and miR-21 in macrophage was abolished (Additional file 1: sFig 10A). Furthermore, exosomes induced downregulation of iNOS, PDCD4, and PTEN, as well as upregulation of Arg-1 in the macrophages was also attenuated when the miRNAs were depleted (Fig. 5B and Additional file 1: sFigure 10B). Flow cytometry analysis also demonstrated that the expression of CD206, B7H4, and CD138 upregulated by exosomes was eliminated (Fig. 5C). The concentration of pro-inflammatory cytokines in macrophages that received miRNAs-knockdown exosomes was much higher than macrophages that received normal exosomes. Conversely, the concentration of anti-inflammatory cytokines was found to be much lower in miRNAs-knockdown exosomes treated macrophages (Fig. 5D). Knockdown miR-16 and miR-21 also abolished exosome-mediated CCL20 upregulation (Fig. 5 E) and increased ROS level (Additional file 1: sFig 10C). As expected, when compared to normal exosome treatment, the miRNAs-knockdown exosome-treated macrophages efferocyted less apoptotic cells (Fig. 5F) and induced fewer T_reg_ cells (Fig. 5G). Additionally, depleting miR-425-5p (Additional file 1: sFigure 11A), which also enriched in the exosome, did not erode the effect of the exosome. The exosome-treated macrophages were polarized to a phenotype similar to the unmodified exosome-treated one (Additional file 1: sFigure 11B). Our findings provide evidence that exosomal miR-16 and miR-21 play a critical role in exosome-induced SLE macrophage anti-inflammatory polarization by regulating the PDCD4 and PTEN pathways.

### Exosome-induced macrophage polarization alleviated pristane-induced mouse lupus nephritis by enhancing efferocytosis and IL-17 + Treg cell recruitment

**Fig. 6:**
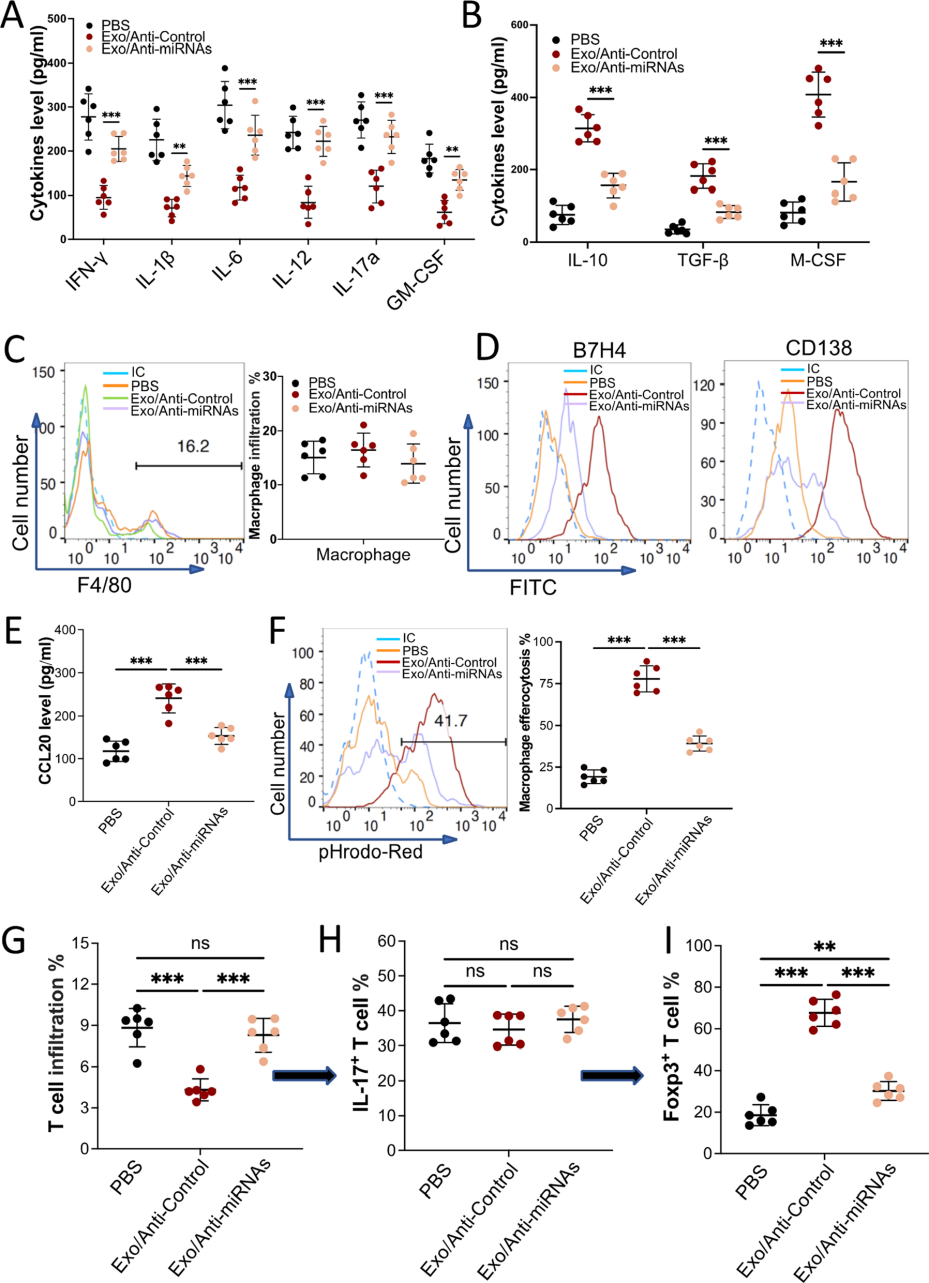
Exosomes from BMMSCs alleviated autoimmunity through polarizing macrophages to a unique M2-like phenotype in a mouse lupus nephritis model. Mouse was peritoneal injected pristane. Four months later, PBS (*n* = 6), Exo/Anti-Control (exosome with normal level of miRNAs, *n* = 6), or Exo/Anti-miRNAs (miR-16- and miR-21-depleted exosome, *n* = 6) were tail vein injected. The mice were euthanized 3 days post-final injection. The blood and kidneys were collected. **A and B** The pro- (**A**) and anti-inflammatory (**B**) cytokines in the mouse serum were checked using ELISA. **C** The F4/80^+^ macrophage infiltration in the kidneys. **D** Macrophage surface markers B7H4 and CD138 were checked by using flow cytometry. **E** The level of chemokine CCL20 secreted from the macrophages was measured using ELISA. **F** The efferocytosis of pHrodo Red-labeled apoptotic TCMK-1 cells by the macrophages. **G–I** The percentage of IL-17^+^ T_reg_ cells in IL-17^+^ T cells was calculated by flow cytometry. The infiltration of CD4^+^ T cell in the kidney from the mice that received various treatment (**G**). The percentage of IL-17^+^ cells in CD4^+^ T cell (**H**). The percentage of Foxp3^+^ cells in IL-17^+^ T cell (**I**). Data are expressed as mean ± SD of *n* = 6, unless specified. **p < 0.01, and ***p < 0.001. ns, nonsignificant. IC, isotype control

To understand the underlying mechanisms of exosomes from BMMSCs in alleviating SLE under pathophysiological conditions, C57BL/J mice were subjected to intraperitoneal injections with pristane to induce mouse lupus nephritis. Four months post-injection was defined as day 0. PBS, 100 µg exosomes from BMMSCs that were transduced with lenti-anti-miR-Cont (Exo/Anti-Control) or exosomes from BMMSCs that were transduced with lenti-anti-miR-16 and lenti-anti-miR-21 (Exo/Anti-miRNAs) were tail vein injected into the mouse on day 0, day 3, and day 7. On day 10, the kidneys, spleen, and blood were harvested from all mice. Our results showed that exosome treatment reduced pro-inflammatory cytokines such as IFN-*γ*, IL-1*β*, IL-6, IL-12, IL-17a, and GM-CSF expression (Fig. 6A) but elevated anti-inflammatory cytokines such as IL-10, TGF-*β*, and M-CSF expression (Fig. 6B) in mouse serum. Knockdown of miR-16 and miR-21 in the exosomes obliterated the anti-inflammatory effect of exosomes. Interestingly, there were no significant differences in kidney macrophage infiltration among the three groups (Fig. 6C). However, the phenotype of infiltrated macrophages was drastically different. The expression of CD206 (Additional file 1: sFig 12A), Arg-1 (Additional file 1: sFig 12B), B7H4, and CD138 (Fig. 6D) were promoted, while the expression of CD86 and iNOS were decreased in the macrophages that collected from Exo/Anti-Control treated mouse kidney. The secretions of macrophages were also shifted, pro-inflammatory cytokines (Additional file 1: sFig 13A) and ROS (Additional file 1: sFig 13B) were downregulated but anti-inflammatory cytokines (Additional file 1: sFig 13C) and chemokine CCL20 (Fig. 6E) were upregulated in mice injected with the Exo/Anti-Control (Control). Efferocytosis assay further confirmed that macrophages collected from Exo/Anti-Control treated mouse kidneys ingested more apoptotic cells (Fig. 6F). Again, depleting miR-16 and miR-21 in the exosome attenuated the effects of exosomes. It has been well established that T cells dominate the development of SLE. Our results revealed that the infiltration of CD3^+^CD4^+^ pan T cells was decreased in the kidney of mice that received Exo/Anti-Control injection (Fig. 6G). Surprisingly, the percentages of IL-17^+^ T cells in pan T cells were similar among all three groups (Fig. 6H). Considering that IL-17^+^ T cells can be divided into two groups: (1) Th 17 cells which are verified to accelerate the progression of SLE, and (2) IL-17^+^ T_reg_ cells which are IL-17^+^ and Foxp3^+^ and thought to contribute to the revitalizing of SLE. Our data demonstrated that in the Exo/Anti-Control treated mouse kidney, most (> 70%) IL-17^+^ T cells were Foxp3^+^ and IL-17^+^ T_reg_ cells. Moreover, our data showed the IL-17^+^ T_reg_ cells infiltrated in the kidney were CCR6^+^ (Additional file 1: sFig 14). In support of our previous data, we demonstrated that macrophages can be polarized by exosomes and secrete a large amount of CCL20, a ligand of CCR6. It is reasonable to postulate these IL-17^+^ T_reg_ cells were recruited or differentiated by exosome polarized macrophages. Moreover, Exo/Anti-Control treatment also increased IL-17^+^ T_reg_ cells in the mouse spleen, compared to Exo/Anti-miRNAs (Additional file 1: sFig 15). The CCR6^+^ IL-17^+^T_reg_ cells enrichment was significantly impaired when using miRNAs depleted exosome. Our in vivo data revealed that exosome injection reduced the inflammatory response in an induced mouse lupus nephritis model by polarizing macrophages to an anti-inflammatory phenotype. These specific polarized macrophages alleviated pristane-induced mouse lupus nephritis by secreting anti-inflammatory cytokines, engulfing apoptotic cells, and recruiting/inducing more IL-17^+^ T_reg_ cells.

## Discussion

The contribution of the innate immune system to the development of SLE has been extensively investigated [21]. In the early stages of SLE, myeloid cells play a pivotal role in the activation of the adaptive immune system by evoking the activation of effector T cells and the production of autoantibodies, which subsequently results in organ damage. In our study, we demonstrate that exosomes from BMMSCs revive SLE mice by promoting high efferocytosis activity and anti-inflammatory polarization of macrophages.

Accumulation of apoptotic cells is thought to be one of the most fatal contributors of SLE [22]. Although the clearance of apoptotic cells is a conserved function that does not require immune stimulation, when the number of apoptotic cells exceeds the clearance capacity of the immune system, the accumulated apoptotic cells can attract immune responses [9, 23]. Undisposed apoptotic debris exposes cellular materials, including nuclear antigens, to the immune system and stimulates an inflammatory response by activating nucleic acid recognition receptors, such as the Toll-like receptor family (TLRs) [24]. Immune cells that express TLRs, including macrophages, dendritic cells, some T cells, and B cells, are activated and can produce many pro-inflammatory cytokines. These cytokines further activate immune cells and lead to the production of autoantibodies as well as decreasing of T_reg_ cells [25, 26]. Restoring the balance between apoptotic cell production and disposal of apoptotic debris is paramount to the treatment of SLE. Macrophages play a key role in the clearing of apoptotic cells in our body. To avoid an inflammatory response, macrophages engulf apoptotic debris in a non-inflammatory way and eliminate antigen-presenting cells which provoke the activation of T cells and B cells. The decreasing efferocytosis activity of macrophages in SLE patients and animal models has been observed [27, 28]. It has also been appreciated that macrophage pro-inflammatory polarization in SLE patients accelerates the progression of SLE [29]. Similarly, in our study the macrophages collected from the kidney of MRL/*lpr* mice exhibit a pro-inflammatory phenotype and lower efferocytosis activity when compared to the macrophages from wild-type mice.

Although mesenchymal stromal cells have been applied to treat SLE and achieved some success, high immunogenicity and costliness are the two major disadvantages that need to be conquered. Exosome therapy, a cell-free therapeutic approach, has evolved for decades to address these issues. Exosomes from stem cells are employed to treat some immune diseases by modulating the immune responses. Exosomes from adipose-derived stem cells are described to induce macrophage anti-inflammatory polarization and therefore attenuate mouse adipose inflammation and mediate white adipose beiging [30]. Exosomes from bone marrow-derived mesenchymal stromal cells induce macrophage tissue repair polarization and promote mouse tendon angiogenesis and healing [31]. We, for the first time, provide evidence that exosomes from BMMSCs alleviate SLE-related nephritis by modulating macrophage phenotype in SLE mice. Compared to PBS-treated macrophages, exosome-treated macrophages were polarized to an anti-inflammatory phenotype, which is defined by the high level of CD206, Arg-1, and anti-inflammatory cytokines. More interestingly, the expression of B7H4 and CD138 is upregulated on exosome-treated macrophages. B7H4, a B7 family molecule, negatively regulates T cell immune response [32], and CD138 is reported to be positively associated with the efferocytosis activity of macrophages [33]. Our in vivo study further demonstrates that the exosomes relieve pristane-induced mouse lupus nephritis by polarizing macrophages to a B7H4^+^CD138^+^ anti-inflammatory phenotype which function to clear apoptotic cells and induce more T_reg_ cells.

MiRNAs in exosomes play an essential role in mediating exosome functions [34]. BMMSCs are recently reported to induce macrophage anti-inflammatory polarization through exosomal miRNAs [35, 36]. Our microarray data shows that eight macrophage polarization-associated miRNAs fluctuate more than two times the amount when compared to exosome-treated macrophages in the control. In our exosome analysis, we found that miR-16 and miR-21 were greatly increased and contributed to the anti-inflammatory polarization of macrophages. MiR-16 is reported to target PDCD4 to suppress macrophage pro-inflammatory polarization in atherosclerosis [37]. MiR-21directly targets PTEN to induce macrophage anti-inflammatory polarization [17]. In this study, overexpression of miR-16 and miR-21 in the macrophages from lupus mice polarized the macrophages to an anti-inflammatory phenotype similar to the exosome-treated macrophage. However, treating macrophages with miR-16- and miR-21-depleted exosomes fail to shift the macrophage polarization. The exosome-treated macrophages maintain high pro-inflammatory cytokines secretion, low efferocytosis activity, and less T_reg_ cell recruitment.

T helper cells, especially Th17 cells, are a crucial contributor to SLE development by secreting pro-inflammatory cytokines [38]. Multiple studies have verified the presence of Th17 cells in SLE patients and lupus mice [39]. The role of IL-17 which is secreted by Th 17 cells, in the development of autoimmunity and pathology in SLE is demonstrated [40]. While T_reg_ cells which are identified as a critical counter of T helper cells, alleviate SLE by conciliating inflammation in SLE patients [41]. The recently identified IL-17^+^ T_reg_ cells are reported to offset the pathogenic Th17 responses in murine SLE. [42] Our study shows that in the pristane-induced mouse lupus nephritis model, exosome treatment results in more IL-17^+^ T_reg_ cells. The increased secretion of CCL20, the only known ligand of CCR6 which is highly expressed on the IL-17^+^ T_reg_ cells, from exosome-treated macrophages is likely to be the key of the IL-17^+^ T_reg_ cell elevation in the SLE mouse model. However, a definite link between exosome primed macrophages and IL-17^+^ T_reg_ cells and the potential underlying mechanisms remain to be established.

## Conclusion

Taken together, our results reveal that exosomes derived from BMMSCs induce a specific macrophage polarization which is defined by a high level of CD206, B7H4, CD138, Arg-1, CCL20, and anti-inflammatory cytokines secretion, in vitro and in vivo. These macrophages could alleviate SLE by eliminating apoptotic debris in a highly efficient way and inducing more T_reg_ cells. Exosomal delivery of miR-16 and miR-21 contributes to the polarization of the macrophage by targeting PDCD4 and PTEN, respectively. This study introduces exosomes as a new therapeutic strategy for autoimmune diseases and expands our vision of the coordination of macrophage and T_reg_ cells in the SLE treatment. The effect of the exosomes on the transition of pro-inflammatory macrophage to anti-inflammatory phenotype may also imply their broader application for other inflammatory-related disease treatments.

## Supplementary Information

**Additional file 1:** Supplementary figures.

## Data Availability

The data that support the findings of this study are available from the corresponding author upon reasonable request.
