# Supplementary material for: Mesenchymal stem cell-derived exosome-educated macrophages alleviate systemic lupus erythematosus by promoting efferocytosis and recruitment of IL-17+ regulatory T cell
Source: Stem Cell Res Ther. 2022 Sep 24;13:484. doi: 10.1186/s13287-022-03174-7 (PMC9509559; doi:10.1186/s13287-022-03174-7)
Supplement: Supplementary file 1 — Additional file 1: Supplementary figures. [file 13287_2022_3174_MOESM1_ESM.pdf]

Mesenchymal stem cell-derived exosome-educated macrophages alleviate systemic lupus erythematosus by promoting efferocytosis and recruitment of IL-17$^+$ regulatory T cell

- **Authors:** Mingchao Zhang[a,c,+], Takerra K. Johnson-Stephenson[d,e,+], Weiran Wang[a], Yang Wang[e,f], Jing Li[a], Limin Li[b], Ke Zen[a,b], Xi Chen[a], Dihan Zhu[b,e].

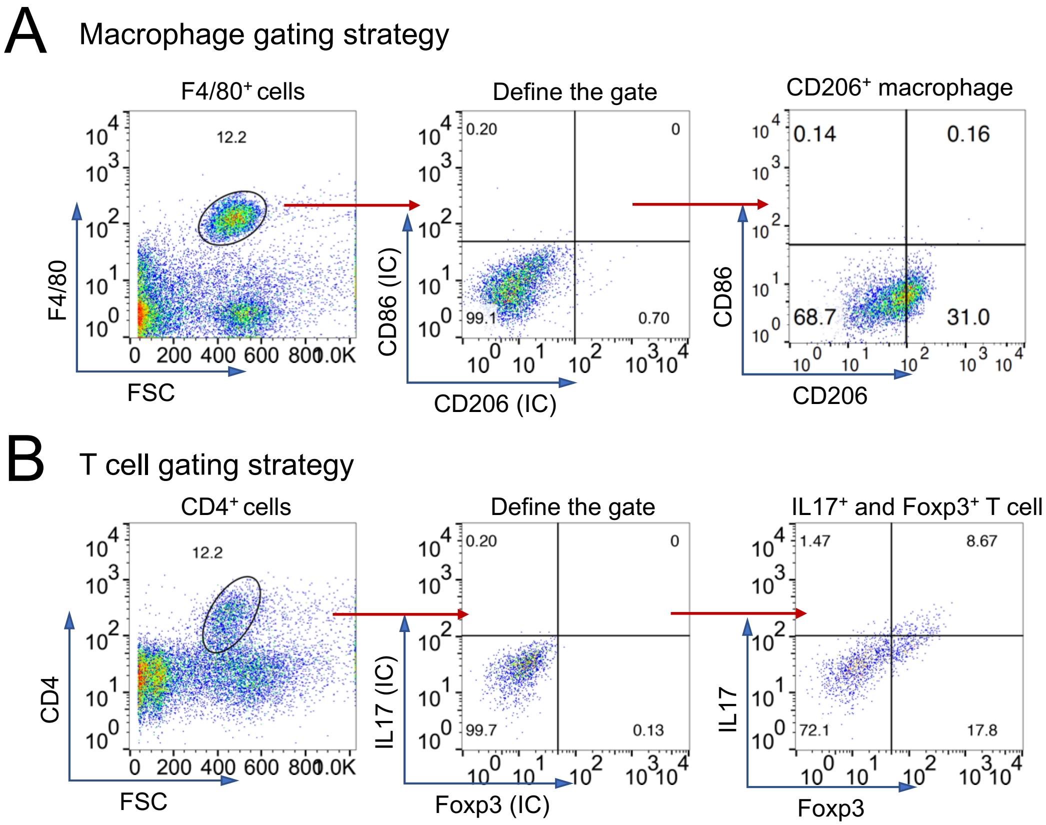

sFigure 1. A. For macrophage gating: FITC-labeled F4/80 were used to gate pan macrophage from a cell mixture. Isotype control was used as the negative control to define the size and position of the gate. The gate was applied to the samples to figure out the phenotype of the macrophages.

B. For T cell gating: FITC-labeled CD4 were used to gate pan $CD4^+$ T cell from a cell mixture. Isotype control was used as the negative control to define the size and position of the gate. The gate was applied to the samples to estimate the percentage of the $IL17^+$ and $Foxp3^+$ T cell. IC, isotype control.

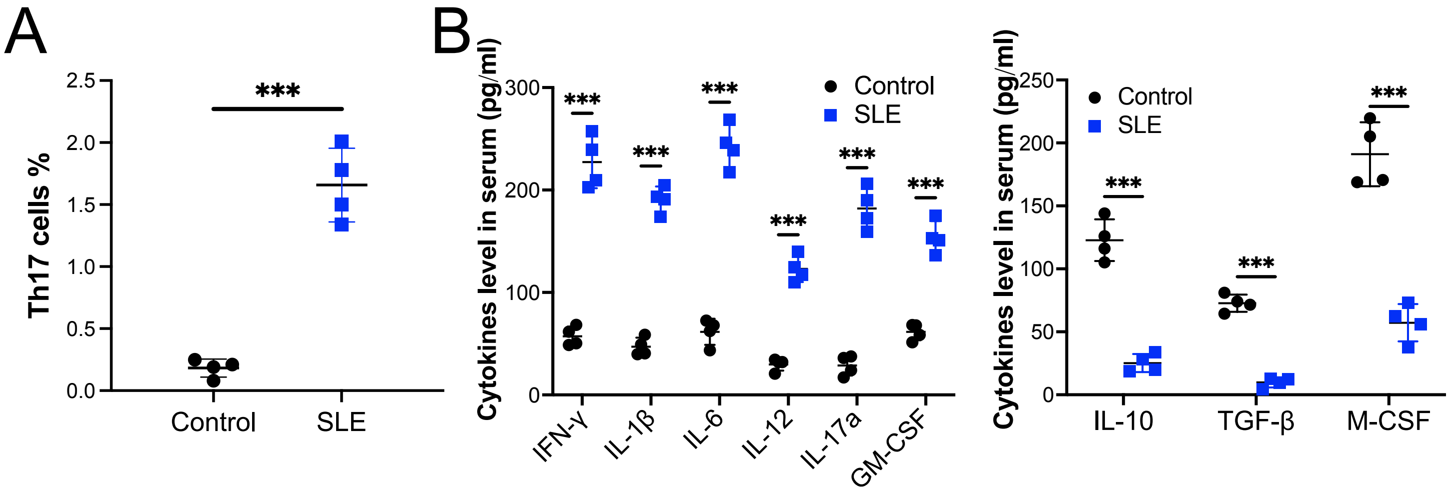

sFigure 2. Th17 cells contributed to the development of SLE. **A,** Spleens of MRL/lpr mice (SLE) were collected. The infiltration of Th17 cells was analyzed by flow cytometry. **B,** The pro-inflammatory and anti-inflammatory cytokines in the serum of MRL/lpr mice were examined. C57BL/6 mice (Control) were used as a control. Data is expressed as mean ± SD of n=4. ***p<0.001.

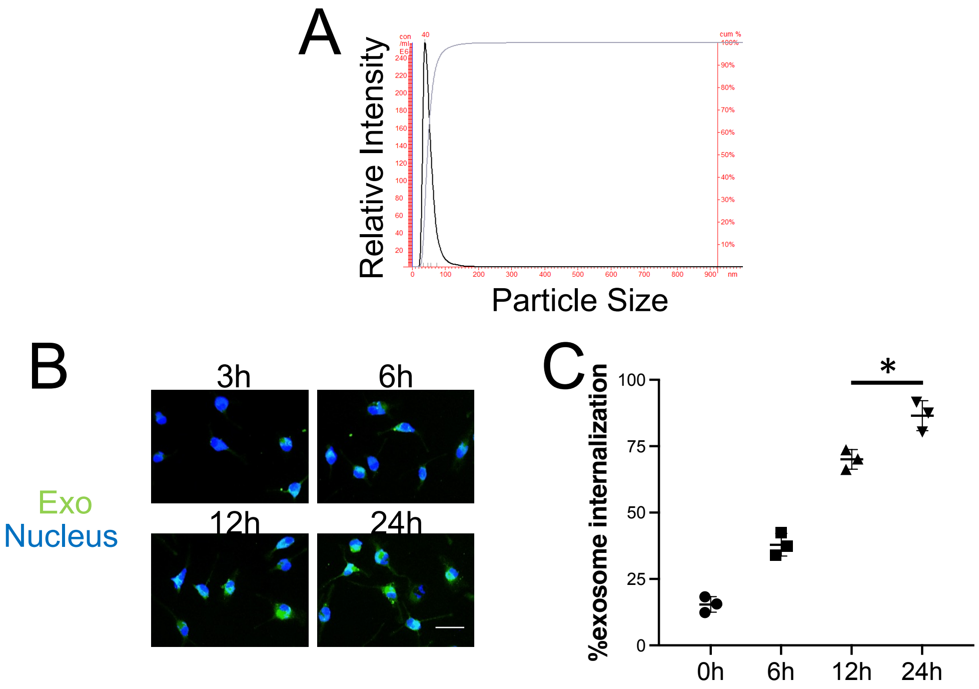

sFigure 3. Characterization of Exosomes from BMMSCs. **A,** The size and intensity of isolated exosomes were examined using nanoparticle tracking analysis (NTA). **B and C,** The uptake of the exosomes into macrophages. The macrophages were incubated with Exo-Green-labeled exosomes at a concentration of 50 μg/ml for the indicated times (C, scale bar: 50 μm). Merged images display Exo (green) for labeled exosomes and Necleus (blue) for nuclei. The internalization of exosomes into macrophages was determined by using flow cytometry (D). Data is expressed as mean ± SD of n=3. *p<0.05.

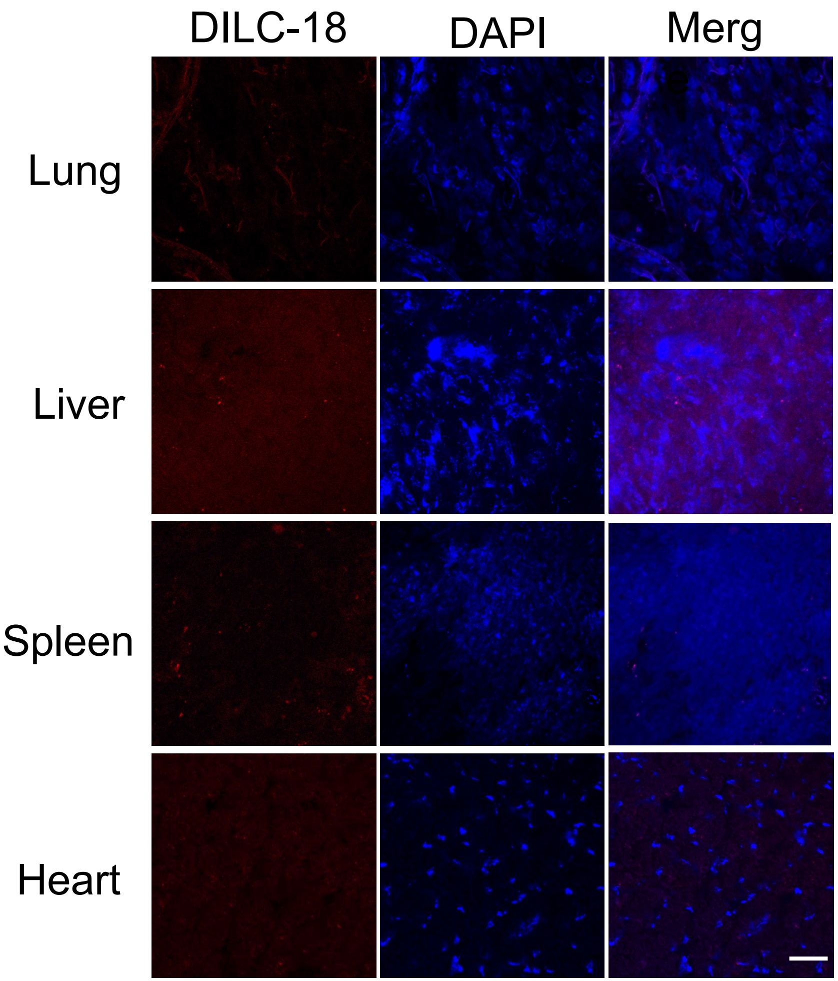

sFigure 4. Distribution of tail vein injected exosomes in mouse. C57BL/6 mice were tail vein injected 100µg DILC-18 labeled exosomes (n=4), 6h post injection, all mice were sacrificed, and the kidney, lung, liver, spleen and heart were collected to test the distribution of the exosomes. Merged images display DILC-18 (red) for labeled exosomes and DAPI (blue) for nuclei (scale bar: 100 µm).

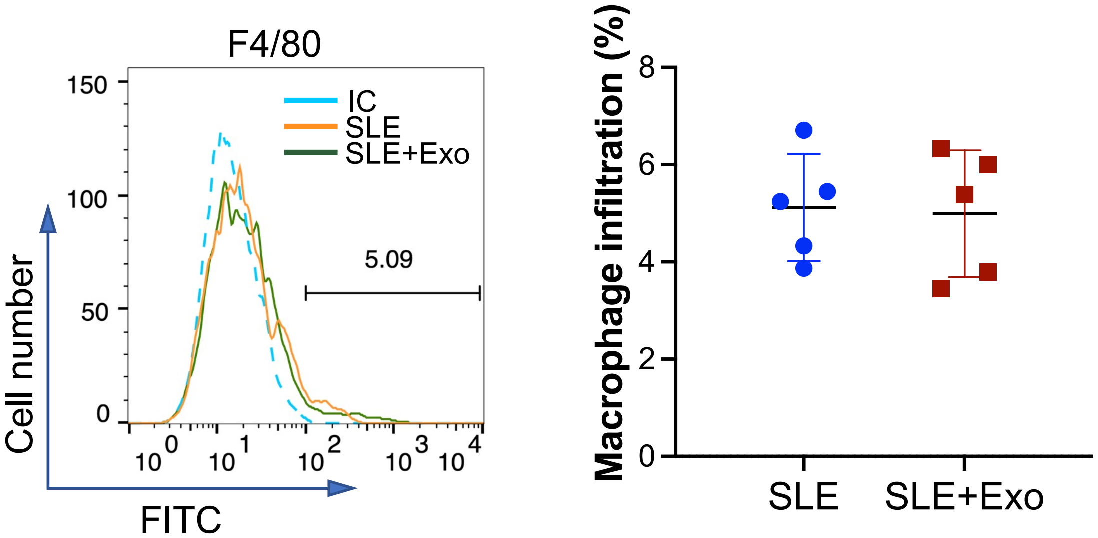

sFigure 5. Macrophage infiltration in the kidney of exosome treated 18-week-old MRL/*lpr* mice. Mice were tail vein injected 100µg BMMSCs derived exosomes at day 0, day 3, and day 7 (SLE+Exo), PBS were used as control (SLE). On day 10, the kidneys were harvested, and the macrophage infiltration were checked. IC, isotype control. Data is expressed as mean ± SD of n=5.

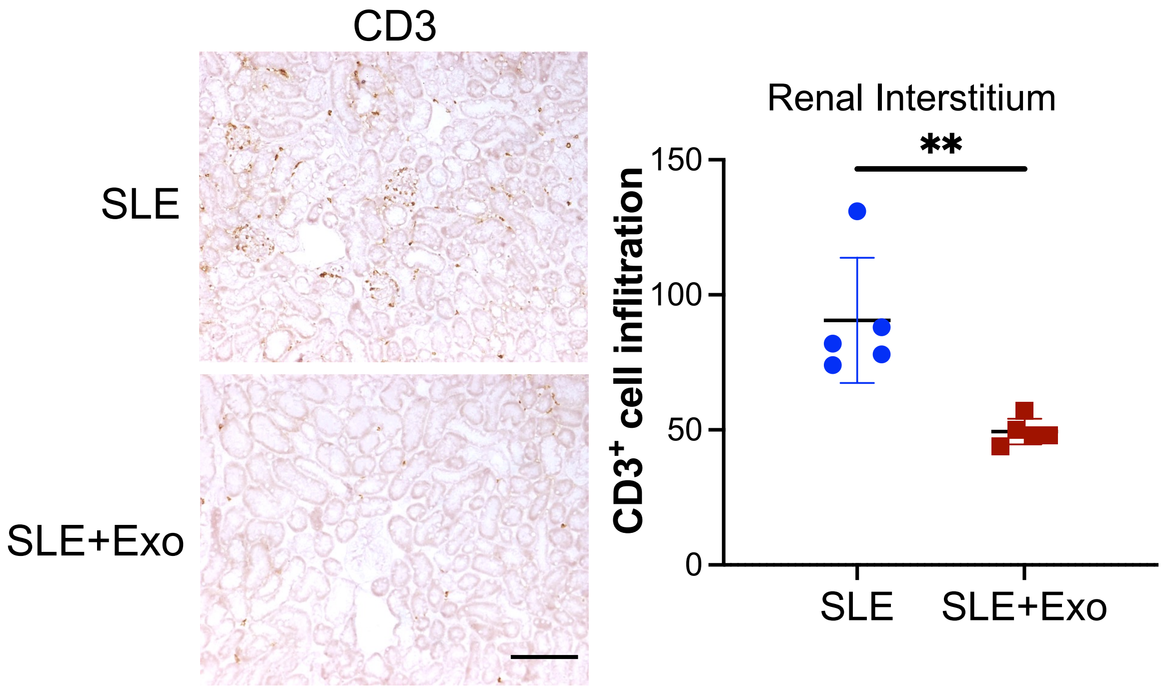

sFigure 6. CD3$^+$ T cell infiltration in the kidney of exosome treated 18-week-old MRL/*lpr* mice. Mice were tail vein injected 100µg BMMSCs derived exosomes at day 0, day 3, and day 7 (SLE+Exo), PBS were used as control (SLE). On day 10, the kidneys were harvested, and the T cell infiltration were checked. T cell infiltration in renal interstitium (scale bar: 100 µm). Data is expressed as mean ± SD of n=5. **p<0.01.

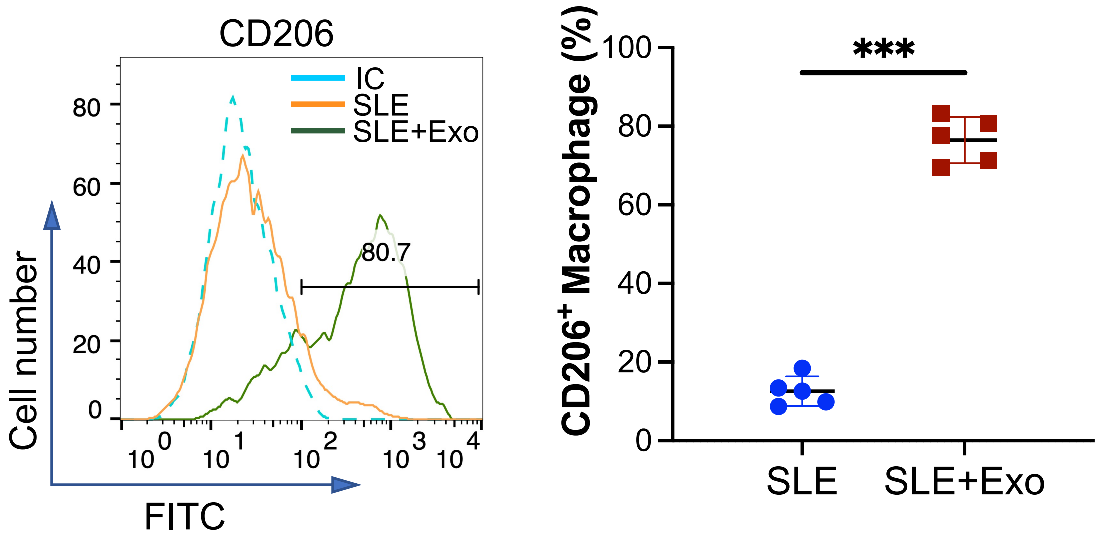

sFigure 7. The percentage of CD206^{+} macrophage in pan kidney infiltrated macrophages from exosome treated 18-week-old MRL/*lpr* mice. Mice were tail vein injected 100μg BMMSCs derived exosomes at day 0, day 3, and day 7 (SLE+Exo), PBS were used as control (SLE). On day 10, the kidneys were harvested, and the percentage of CD206^{+} macrophage were checked. IC, isotype control. Data is expressed as mean ± SD of n=5. ***p<0.001.

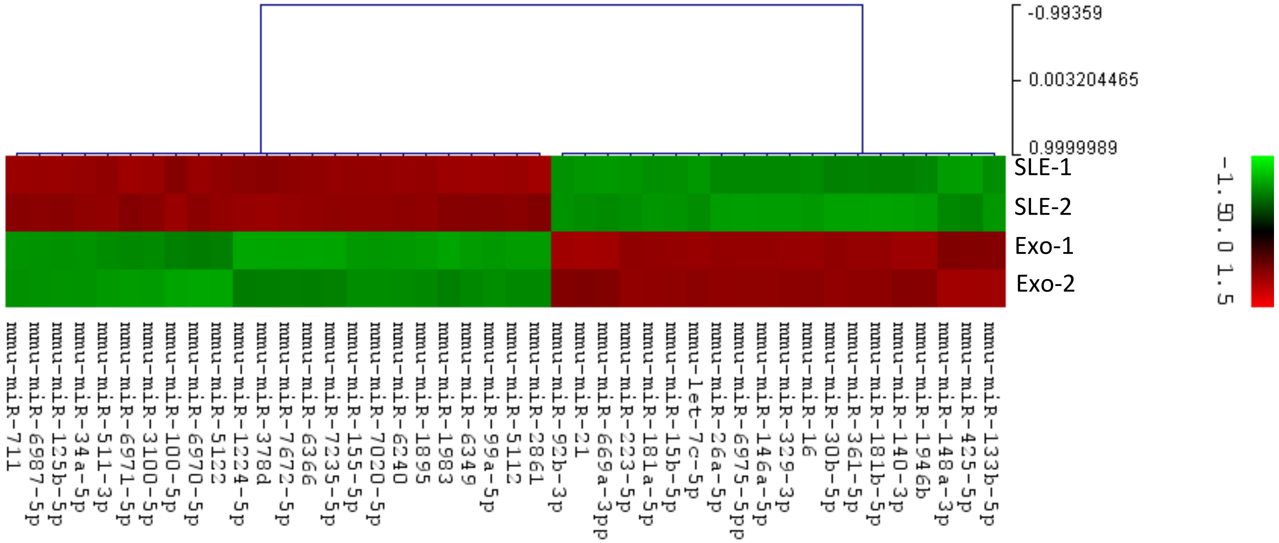

sFigure 8. Profiling of miRNAs in macrophages from MRL/lpr mice that received PBS (SLE) or exosomes (Exo). Heat map showed the miRNAs only with the difference between SLE and Exo (n = 2).

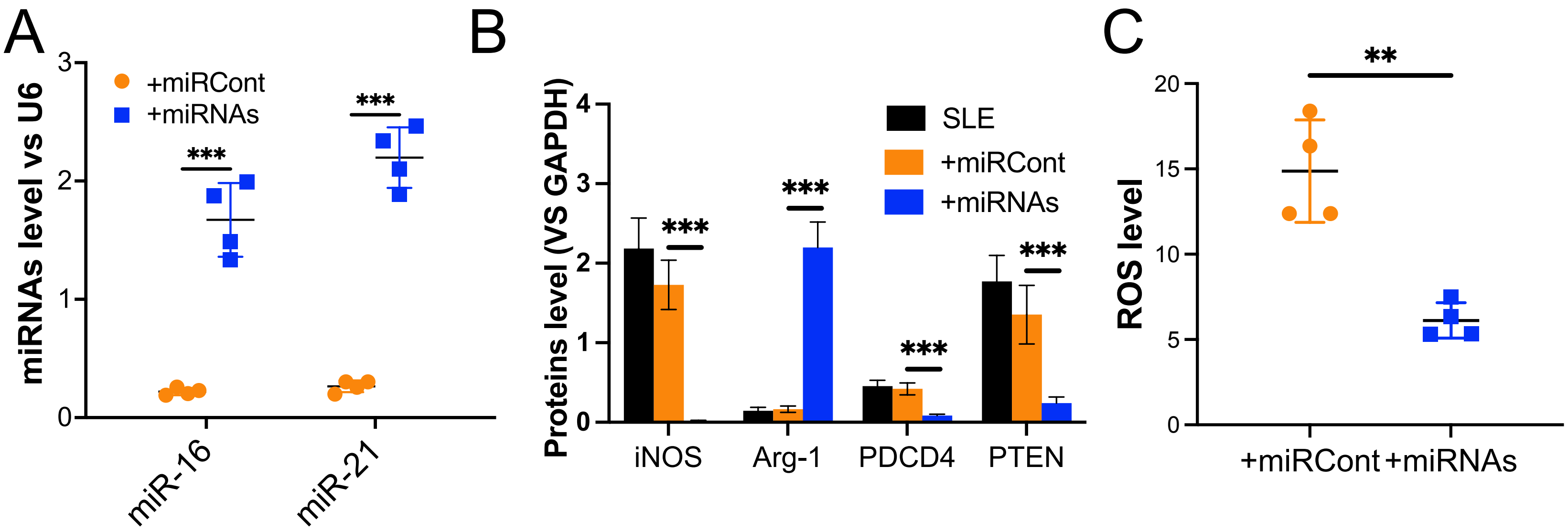

sFigure 9. MiR-16 and miR-21 regulated macrophage polarization. Macrophages from the kidney of MRL/lpr mice were transduced with lentivirus-based miR-16 and miR-21 overexpression vector (+miRNAs). The miRNA-Lentivirus control was used as a control (+miRCont). **A,** The level of miR-16 and miR-21in the macrophages. U6 was used as an internal control. **B,** The statistical analysis of the density of the immunoblotting bands using Image J software (n = 3). **C,** The ROS level of the macrophages. Data is expressed as mean ± SD of n=4. **p<0.01 and ***p<0.001.

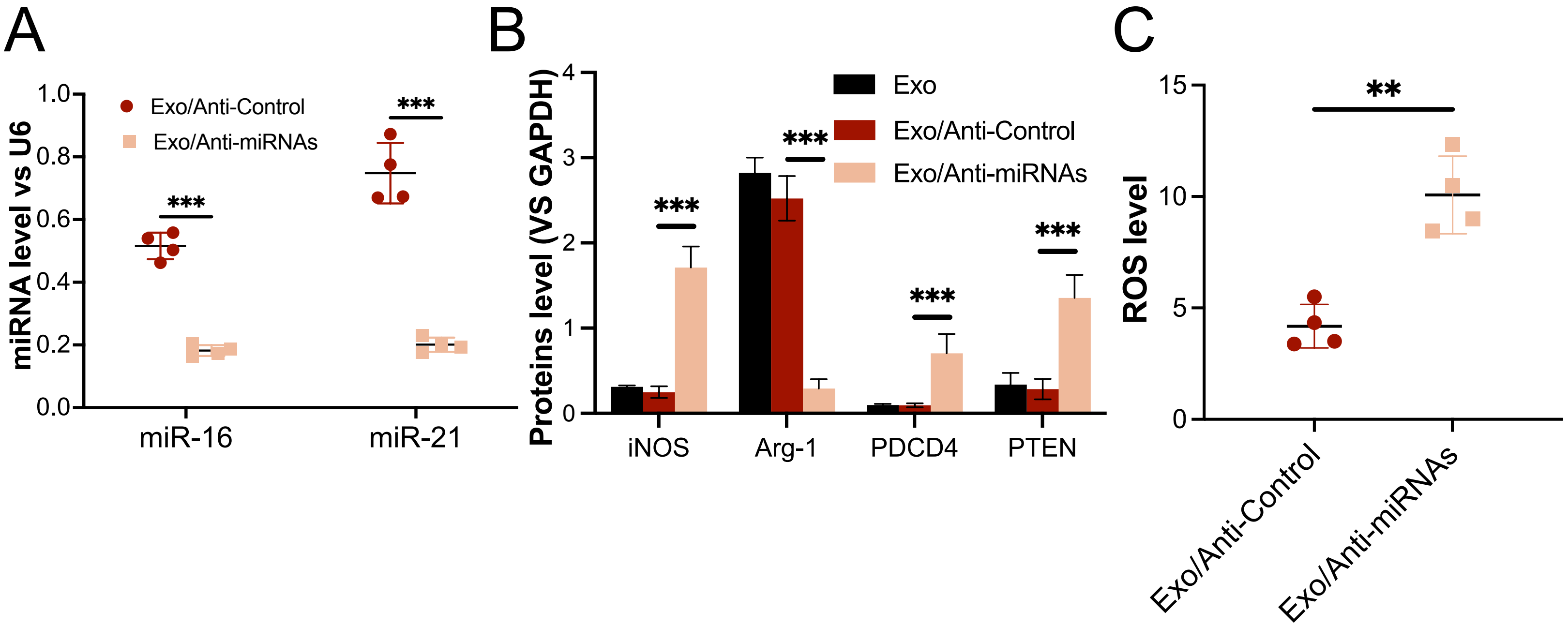

sFigure 10. MiR-16 and miR-21 contributed to the exosome induced macrophage polarization. Macrophages from the kidney of MRL/lpr mice were treated with Exo/Anti-Control (exosome with normal miRNAs' level) or Exo/Anti-miRNAs (miR-16 and miR-21 depleted exosome). **A,** The level of miR-16 and miR-21in the macrophages. U6 was used as an internal control. **B,** The statistical analysis of the density of the immunoblotting bands using Image J software (n = 3). **C,** The ROS level of the macrophages. Data is expressed as mean ± SD of n=4. **p<0.01 and ***p<0.001.

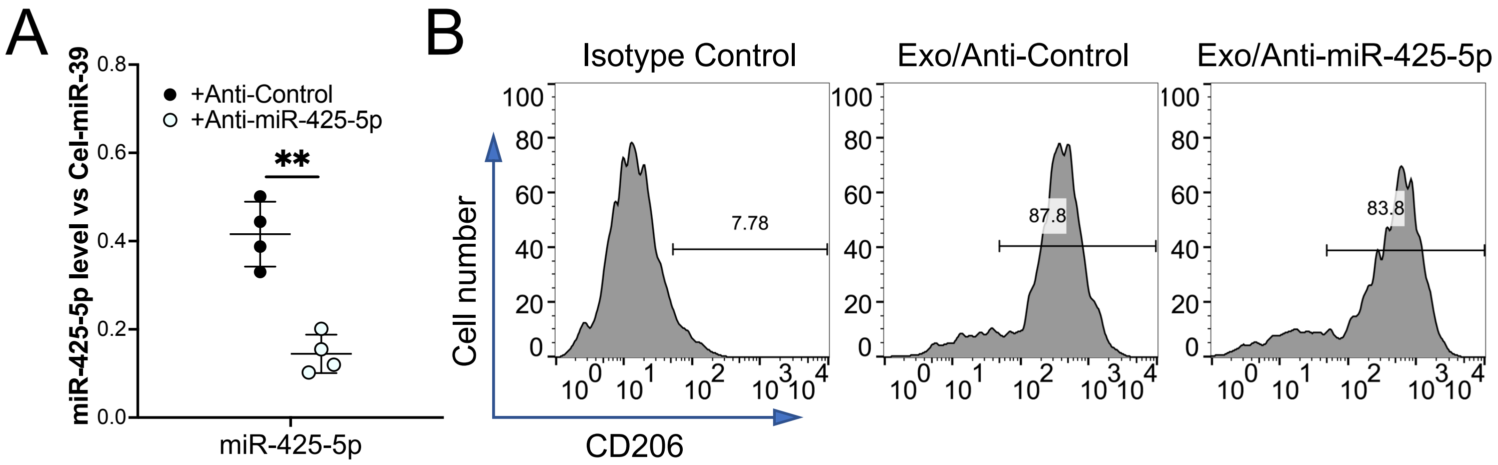

sFigure 11. Depleting miR-425-5p did not attenuate pro-anti-inflammatory polarization effect of the exosome. Macrophages from the kidney of MRL/lpr mice were treated with miR-425-5p depleted exosomes (Exo/Anti-miR-425) or Exo/Anti-Control. **A,** The level of miR-425-5p in the exosome. Cel-miR-39 was used as an exogenous reference. **B,** The percentage of $CD206^+$ macrophages in the macrophages that received various treatments. Data is expressed as mean ± SD of n=4. **p<0.01.

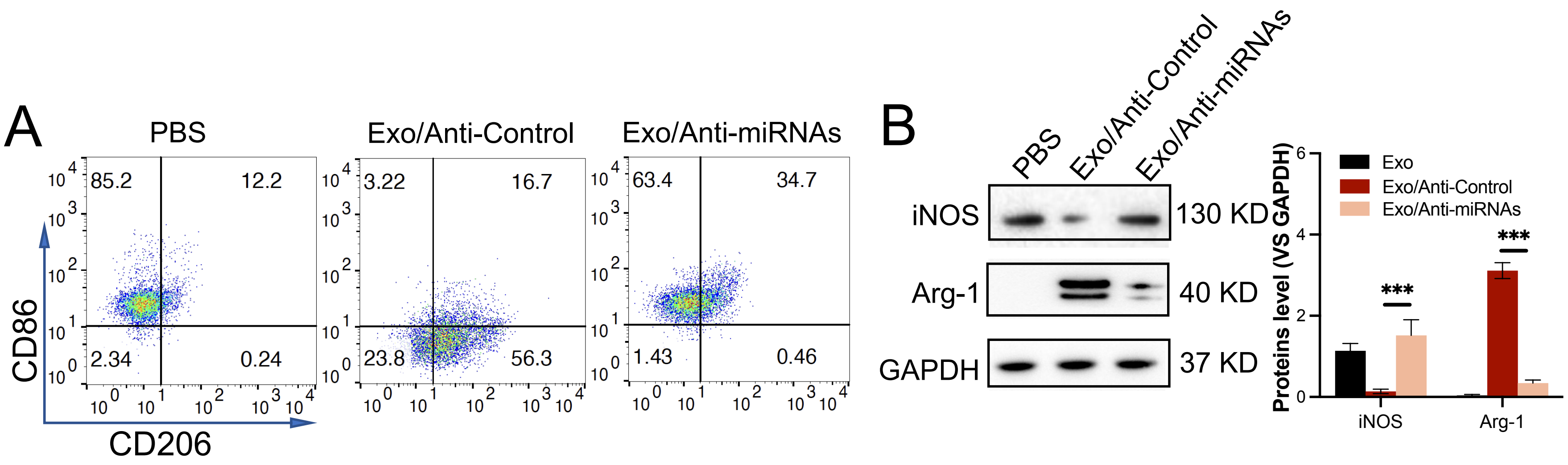

sFigure 12. Exosome modulated macrophage polarization in pristane induced lupus nephritis mice. The mice were injected with PBS, Exo/Anti-Control or Exo/Anti-miRNAs. Macrophages in the kidney of the mice were collected. **A,** The percentage of CD86$^+$ or CD206$^+$ macrophages (n=6). **B,** The level of iNOS and Arg-1 in macrophages (n=4). CAPDH were used as a control. Data is expressed as mean ± SD. ***p<0.001.

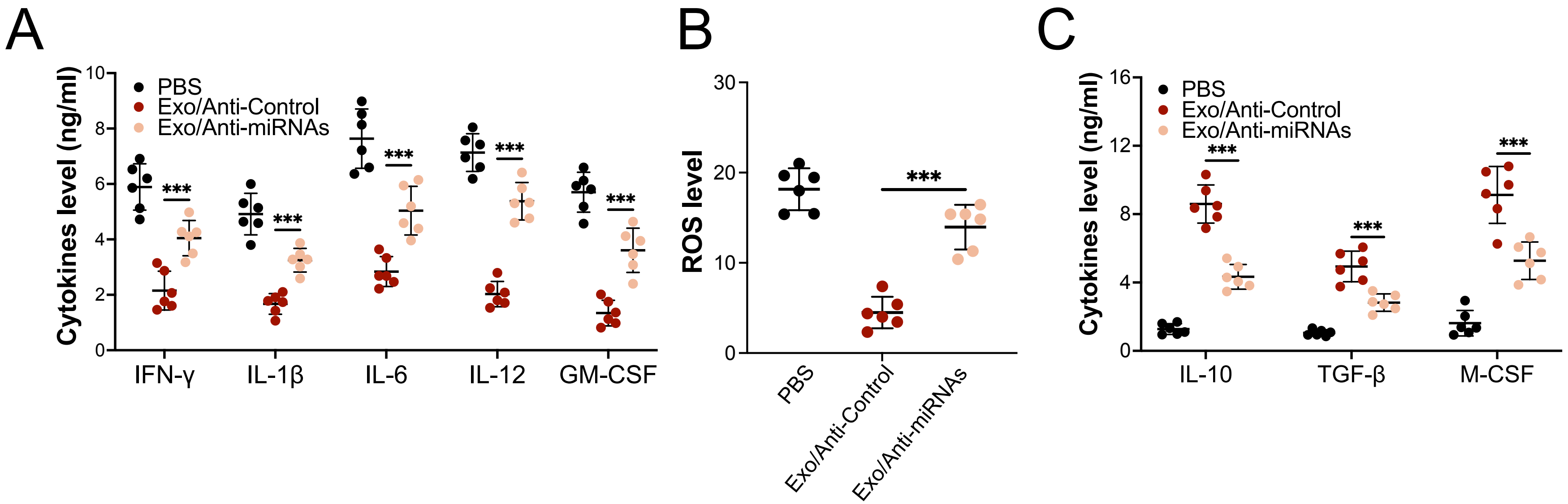

sFigure 13. Exosome shifted macrophage cytokines and ROS secretion. Conditioned medium of macrophages from the kidney of the lupus mice that received PBS, Exo/Anti-Control or Exo/Anti-miRNAs, were collected. Cytokines and ROS level were tested. **A,** The levels of pro-inflammatory cytokines. **B,** The level of ROS. **C,** The levels of anti-inflammatory cytokines. Data is expressed as mean ± SD of n=6. ***p<0.001.

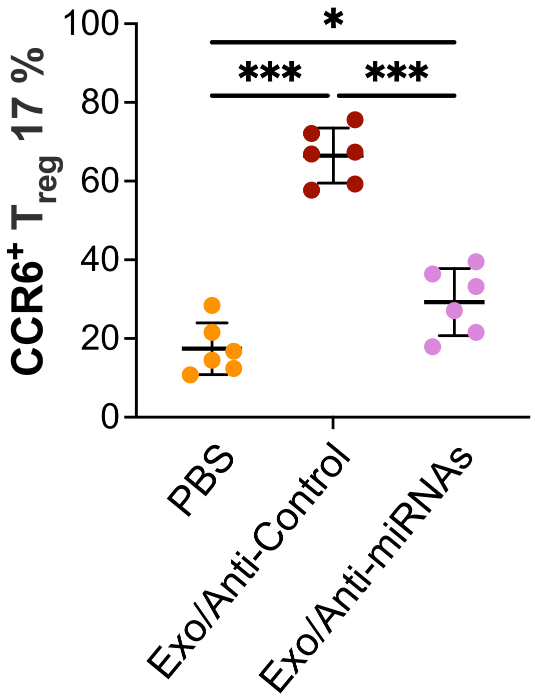

sFigure 14. IL-17^{+} T_{reg} cells were isolated from the kidney of lupus nephritis mice that received PBS, Exo/Anti-Control or Exo/Anti-miRNAs injection. The percentage of CCR6^{+} T_{reg} 17 cells were analyzed. Data is expressed as mean ± SD of n=6. *p<0.05 and ***p<0.001.

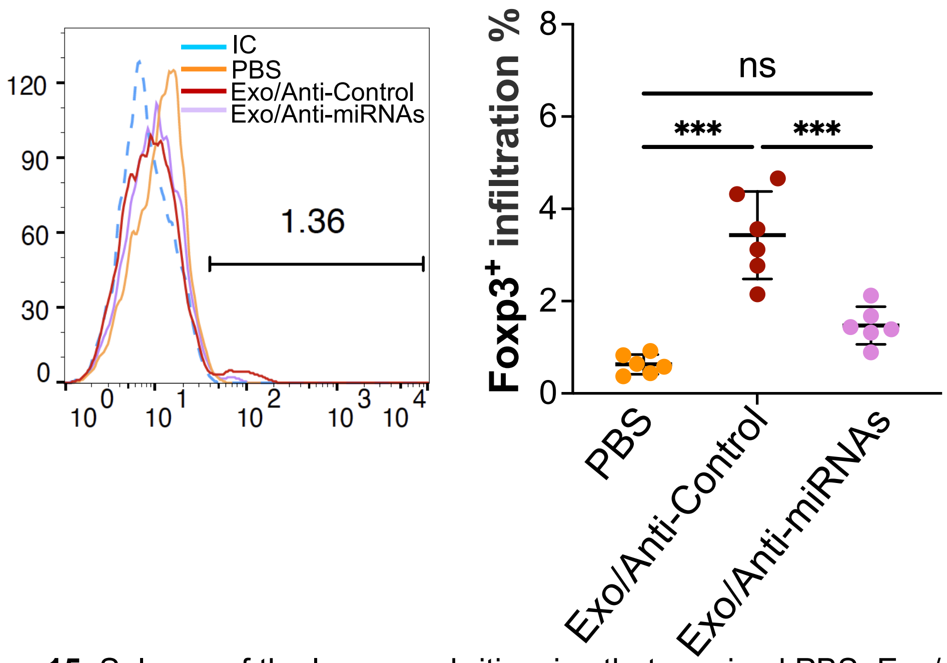

sFigure 15. Spleens of the lupus nephritis mice that received PBS, Exo/Anti-Control or Exo/Anti-miRNAs, were collected. The infiltration of $Foxp3^+$ T cells were examined. Data is expressed as mean ± SD of n=6. ***p<0.001. ns, non-significant. IC, isotype control.

Fig 1D iNOS (130KD)

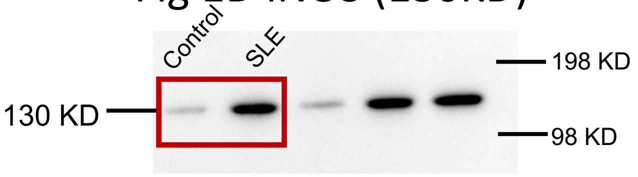

Fig 1D Arg-1 (40KD)

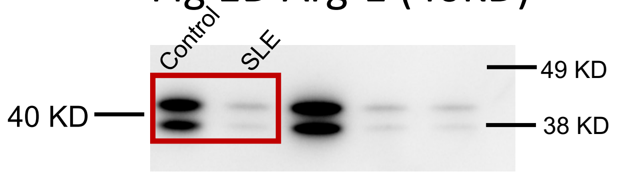

Fig 1D GAPDH (37KD)

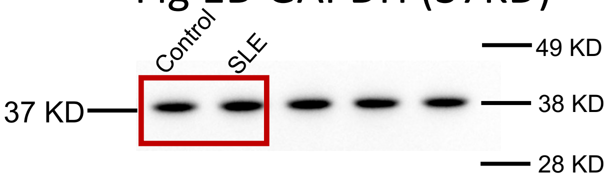

Fig 2A CD9 (42KD)

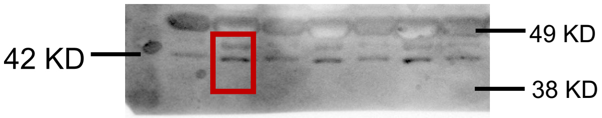

Fig 2A Alix (97KD)

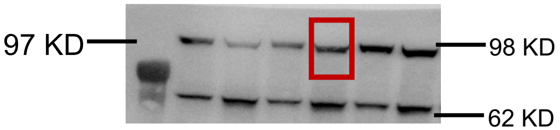

Fig 3C&4C iNOS (130KD)

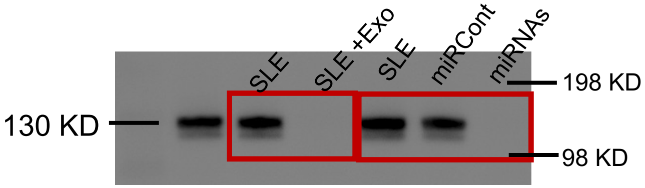

Fig 3C&4C Arg-1 (40KD)

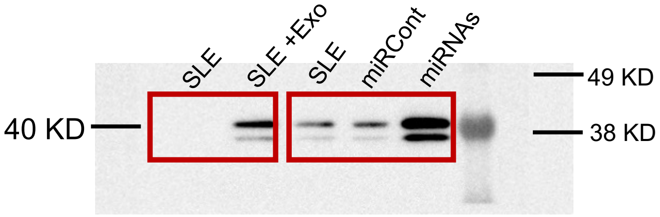

Fig 3C&4C GAPDH (37KD)

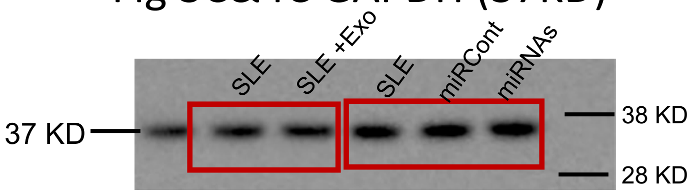

Fig 4C&5B PDCD4 (60KD)

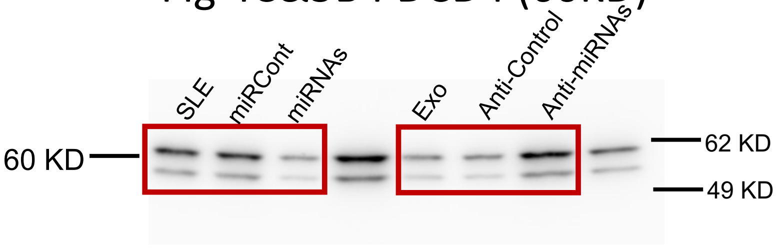

Fig 4C PTEN (54KD)

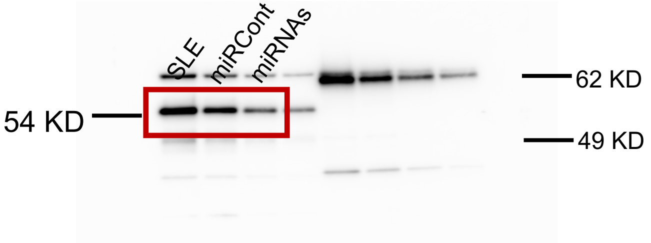

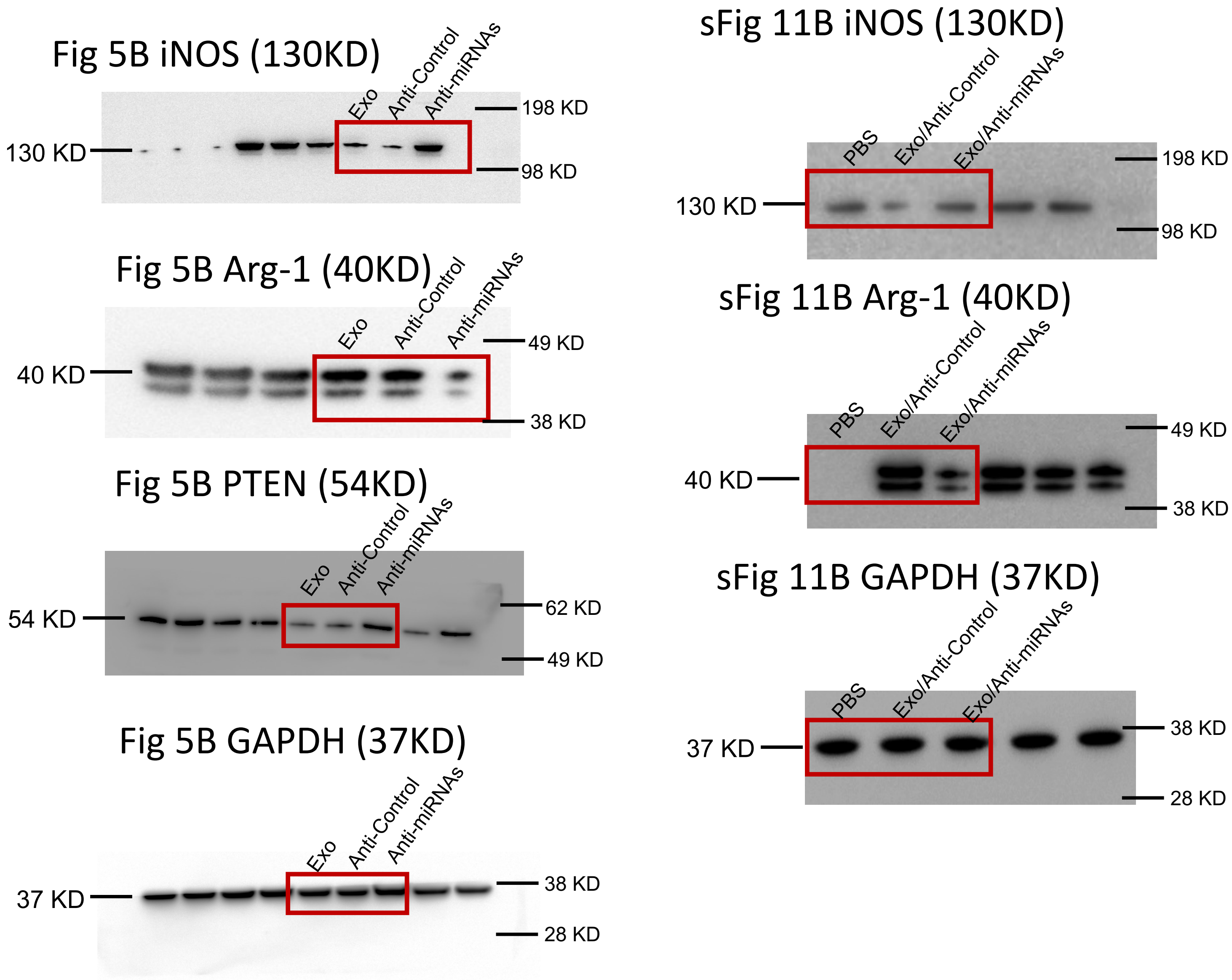
Fig 5B iNOS (130KD)
Exo
Anti-Control
Anti-miRNAs
130 KD
198 KD
98 KD
Fig 5B Arg-1 (40KD)
Exo
Anti-Control
Anti-miRNAs
40 KD
49 KD
38 KD
Fig 5B PTEN (54KD)
Exo
Anti-Control
Anti-miRNAs
54 KD
62 KD
49 KD
Fig 5B GAPDH (37KD)
Exo
Anti-Control
Anti-miRNAs
37 KD
38 KD
28 KD
sFig 11B iNOS (130KD)
PBS
Exo/Anti-Control
Exo/Anti-miRNAs
130 KD
198 KD
98 KD
sFig 11B Arg-1 (40KD)
PBS
Exo/Anti-Control
Exo/Anti-miRNAs
40 KD
49 KD
38 KD
sFig 11B GAPDH (37KD)
PBS
Exo/Anti-Control
Exo/Anti-miRNAs
37 KD
38 KD
28 KD
